# Interpretable Machine Learning of Two‐Photon Absorption

**DOI:** 10.1002/advs.202204902

**Published:** 2023-01-19

**Authors:** Yuming Su, Yiheng Dai, Yifan Zeng, Caiyun Wei, Yangtao Chen, Fuchun Ge, Peikun Zheng, Da Zhou, Pavlo O. Dral, Cheng Wang

**Affiliations:** ^1^ State Key Laboratory of Physical Chemistry of Solid Surfaces Department of Chemistry College of Chemistry and Chemical Engineering, iChem Innovation Laboratory for Sciences and Technologies of Energy Materials of Fujian Province (IKKEM) Xiamen University 361005 Xiamen P. R. China; ^2^ Department of Chemistry College of Chemistry and Chemical Engineering iChem Xiamen University Fujian Provincial Key Laboratory of Theoretical and Computational Chemistry Xiamen University 361005 Xiamen P. R. China; ^3^ School of Mathematical Sciences and Fujian Provincial Key Laboratory of Mathematical Modeling and High‐Performance Scientific Computation Xiamen University Xiamen 361005 P. R. China

**Keywords:** conjugation length, machine learning, rational design, two‐photon absorption

## Abstract

Molecules with strong two‐photon absorption (TPA) are important in many advanced applications such as upconverted laser and photodynamic therapy, but their design is hampered by the high cost of experimental screening and accurate quantum chemical (QC) calculations. Here a systematic study is performed by collecting an experimental TPA database with ≈900 molecules, analyzing with interpretable machine learning (ML) the key molecular features explaining TPA magnitudes, and building a fast ML model for predictions. The ML model has prediction errors of similar magnitude compared to experimental and affordable QC methods errors and has the potential for high‐throughput screening as additionally validated with the new experimental measurements. ML feature analysis is generally consistent with common beliefs which is quantified and rectified. The most important feature is conjugation length followed by features reflecting the effects of donor and acceptor substitution and coplanarity.

## Graphical abstract

Two‐photon absorption (TPA) cross section of organic molecules is predicted by a machine‐learning model with experimental accuracy. The model only adopts four molecular features that have clear physical meanings. The model is also validated by experimental measurements of 16 new TPA molecules.

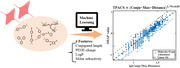

## 1 Introduction

Two photon‐absorption (TPA) is a nonlinear coherent process in which a molecule simultaneously absorbs two photons.^[^ ^1^ ^]^ TPA has been crucial in many technologies, including upconverted laser,^[^ ^2^ ^]^ two‐photon bioimaging,^[^ ^3^ ^]^ two‐photon photodynamic therapy,^[^ ^4^ ^]^ and 3D printing.^[^ ^5^ ^]^ A range of molecules and materials with high TPA cross sections (TPACS, 𝜎) were discovered,^[^ ^6^ ^]^ as determined by the Z‐scan^[^ ^7^ ^]^ and two‐photon excited fluorescence methods.^[^ ^8^ ^]^

A general design principle was established by few‐state models for constructing TPA molecules: creating donor (D)–acceptor (A) push‐pull structure together with a long *π*‐conjugation in the molecule.^[^ ^6a,d^ ^]^ Both features can lead to large transition dipole moments. In addition, quadrupolar D‐*π*‐A‐*π*‐D/A‐*π*‐D‐*π*‐A or multipolar DA*_n_*/AD*_n_* structures are also considered to be beneficial to obtaining large TPACS according to charge resonance models.^[^ ^6^, ^9^ ^]^ However, these observations were made on a limited selection of systems and were not extensively tested considering all the experimental results obtained by the research community over the past years.

High‐accuracy quantum chemical (QC) models can test the validity of these empirical design rules from the first principles.^[^ ^9^ ^]^ However, most QC methods still suffer from poor performance in predicting TPACS,^[^ ^10^ ^]^ and the high‐level QC calculations are usually expensive for examining many molecules with diverse structures and often, of considerably large size.

Machine learning (ML) can complement the QC methods to accelerate materials discovery.^[^ ^11^ ^]^ Here we used an ML approach to study the structure–property relationship of TPA molecules based on reported experimental data containing TPACS of 856 molecules. The goal of our study is to provide a valuable tool for practitioners in the field which can potentially be used both for applied purposes and to answer fundamental scientific questions. Applied purposes include efficient high‐throughput virtual screening (HTVS) to identify lead TPA compounds and designing molecules with high TPACS. Scientific questions which we investigate by using the interpretability of ML are

1. Is there a quantitative relationship between the TPACS and the conjugation length of a molecule ?^[^ ^6a^ ^]^
2. Does a branched DA*_n_* or AD*_n_* structure have an edge over a simple D–A conjugation after eliminating the contribution of elongated conjugation length?^[^ ^12^ ^]^
3. Are there other critical structural features beyond the donor–acceptor, conjugation, and multipolar to determine the TPACS?

## 2 Results and Discussion

### 2.1 Dataset

**Figure 1:**
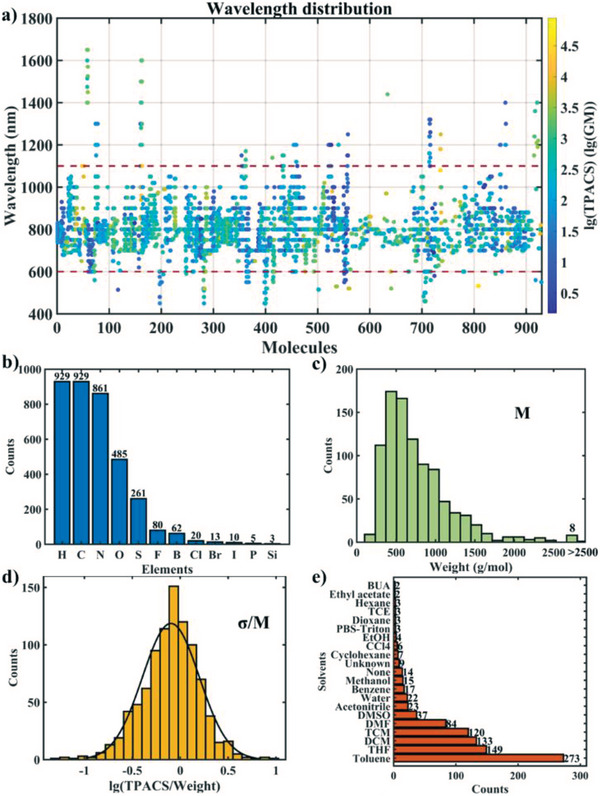
Dataset of TPACS of organic compounds. a) Scatter plot of the distribution of wavelengths at which TPACS was measured; markers are color‐coded according to lg(TPACS). Histograms of b) elements contained in this dataset, c) molecular weight, d) the lg(TPACS) per molecular weight, and e) solvents.

An experimental dataset of 929 unique organic chromophores was collected from 275 literature reports (see Data availability). The dataset contains the TPACSs, the SMILES, names of the molecules, wavelengths of the TPA test, TPA measurement methods, solvents, and DOI number of the source publication. 443 molecules have only one TPACS value measured at a single wavelength, while the remaining 486 molecules have 2–11 TPACS values measured at different wavelengths (**Figure** **1**a). The accuracy of the reported TPACS is difficult to check, but the level of accuracy is partially reflected by comparing the TPACS values of several dyes from different sources^[^ ^13^ ^]^ (Figure S1, Supporting Information), which are 52 ± 41 GM (or 1.7 ± 0.3 in logarithm) for Rhodamine B at 798–802 nm. We use lg(TPACS) in the following studies considering this level of accuracy. In addition, we also put all the molecular features for ML in the datasets, which are described in Section 2.2.

The distribution of the molecular weights is shown in Figure 1c, while the distribution of the logarithm of TPACS per molecular weight is shown in Figure 1d; both are close to the normal distribution. The molecules in the dataset contain many elements, C, H, N, O, S, F, B, Cl, Br, P, Si, and I (in the order of their abundance), but the majority of molecules (564) contains only C, H, N, O (Figure 1b). The count of molecules measured in each solvent is shown in Figure 1e; many of the molecules (273) were measured in toluene, while altogether 21 different solvents were used. In order to avoid inaccuracy due to sparse data near the boundaries, only data points measured at wavelengths from 600 to 1100 nm were used, and the molecules containing P, Si, I elements were eliminated. A dataset containing 856 molecules were used in the following study.

### 2.2 Featurization of Molecules

The wavelengths and solvents used in the TPA measurements were extracted as part of the features. The solvent information is encoded by three descriptors (ET(30), dielectric constant, and dipole moment). The ET(30)^[^ ^14^ ^]^ is defined by electronic transition energy of betaine 30 in different solvents to parameterize effect of solvent polarity. The information of the measurement methods is not used in the ML study, as many entries lack this information.

**Figure 2:**
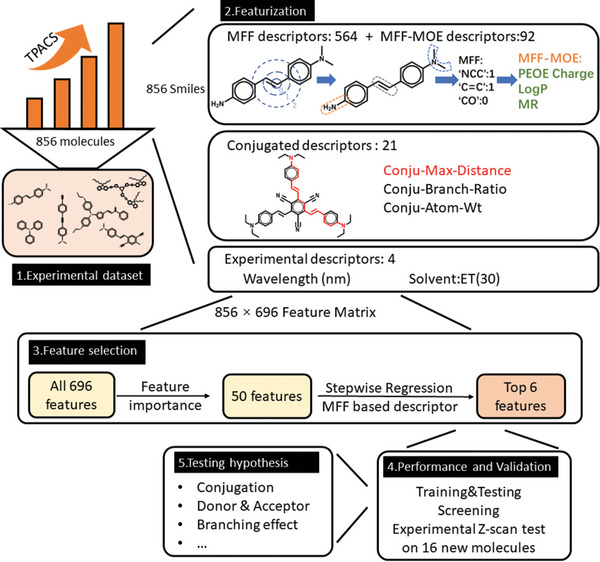
Featurization and feature selection. A scheme explaining molecular fragment fingerprint (MFF) featurization, conjugation features, experimental features, and the procedure of the feature selection. (MFF‐MOE: MFF‐based Molecular Operating Environment features. Conju‐Max‐Distance: The maximum conjugated length in one molecule. Conju‐Branch‐Ratio: a parameter to describe branching in the conjugated system. Conju‐Atom‐Wt: The atomically averaged weight in one molecule).

564 of the features come from molecular fragment fingerprint (MFF) featurization.^[^ ^15^ ^]^ In MFF, molecular fragments were generated by the extended‐connectivity fingerprints (ECFP) method using a radius of 4 supported by the Deepchem python toolkit.^[^ ^16^ ^]^ A vector recording the appearance times of each fragment in a molecule^[^ ^17^ ^]^ was then created (**Figure** **2**). Note that this is different from the unhashed Morgan fingerprints, as the MFF counted fragment structures without considering their further linkage to other parts of the molecule, while the Morgan fingerprints contain this information. The MFF is thus a simplification of the Morgan fingerprints to fit into the needs of analyzing a small dataset. An additional 107 features were generated by the RDKit Python toolkit,^[^ ^17^, ^18^ ^]^ which provide geometrical and electronic structural information of the molecule.

Given the well‐known importance of conjugation for TPACS,^[^ ^19^ ^]^ we also created 21 conjugation features to describe the size, shape, and electronic properties of the conjugation structure (Section S2, Supporting Information). Because electronic properties are best described by quantum mechanics (QM), we also tested a range of QM features that are often used in ML modeling.^[^ ^20^ ^]^ QM features did not lead to significant improvement in accuracy, while their generation is more computationally costly (Section S15, Supporting Information). Hence, we did not consider QM features in the following.

Overall, we obtained 696 initial features (**Table** **1**), all of which have clear physical definitions and can be calculated very fast. More features are introduced in the following sections.

**Table 1:** The features used in this study

Name	Number	Description
Initial features for model screening and feature selection (696)		
MFF	564	Describing molecular structure and functional groups
RDKit	107	Describing molecular shape and electronic structure
Conjugation	21	Describing the properties of conjugation structure
Solvent	3	Describing the polarity of solvents
Wavelength	1	Experimental TPA wavelength in nm
Adding MFF‐based features to enhance interpretability		
MFF‐MOE features	80	Atom‐attributed properties summed up to MFF
Other features for SHAP analysis		
DAratio	1	Distance between donor and acceptor divided by conjugation length

### 2.3 Feature Selection

We assess the importance of these features using three ML models: Least Absolute Shrinkage and Selection Operator^[^ ^21^ ^]^ (LASSO), Gradient Boosting Regression Tree^[^ ^22^ ^]^ (GBRT), and Extreme Gradient Boosting^[^ ^23^ ^]^ (XGBoost) regressor. In the ML process, the datasets were randomly split into the training set and the test set via cross‐validation (CV), and the Mean Squared Error (MSE), Mean Absolute Error (MAE), and R^2^ score of the test sets were calculated to evaluate model performance.

For LASSO, importance of a given feature is manifested by the magnitude of the regression coefficient of the feature. For the GBRT and XGB Regressor, SHAP,^[^ ^24^ ^]^ a Python toolkit to calculate Shapley values, was implemented to generate more interpretable feature importance. We then combined the feature importance indexes of the three regressors (averaged over 240 CV runs) into a weighted one (Section S3 and Figure S2, Supporting Information), which was used to remove the least important features one at a time from the feature matrix.

**Figure 3:**
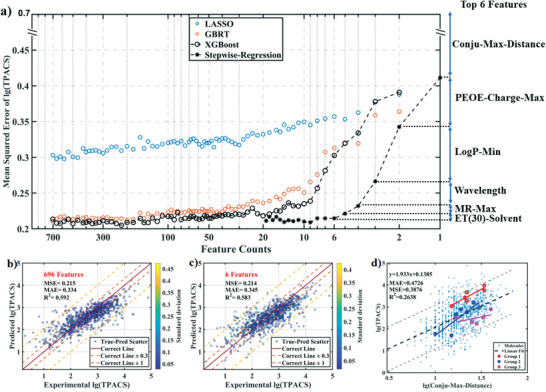
Model performance during feature selection procedure. a) Mean squared error (MSE) against feature selection procedure: feature importance‐based feature selection in LASSO, GBRT, and XGBoost were denoted as blue, red, and black circles, respectively; black stars represent the stepwise regression; top 6 features selected by stepwise regression were shown on the right of the *y*‐axis; the number of features, the metrics and the TPACS are all shown in log scale. Scatter plot of models using b) [856 (number of molecules) × 696 (number of features)] and c) [856 × 6] feature matrices and the XGBoost regressor: the standard deviations of the predicted values in the 240 CV runs of the model is represented by the color axis in log scale. d) The parity plot of experimental lg(TPACS) versus lg(Conju‐Max‐Distance): three groups of structurally related compounds of different conjugation lengths in the dataset are also highlighted by red, blue, and purple circles; note that it is difficult to find examples of homologous structures, and these selected series also differ in functional groups.

Through this deletion process, we obtained 50 features that can retain the performance of the models (**Figure** **3**a; and Table S1, Supporting Information), as shown by the scatter plots of true versus predicted values of testing sets (Figure 3b).

As there were still highly correlated features within the 50 features shown by correlation coefficients matrix (Figure S3, Supporting Information), we further reduced the number of features by stepwise regression. The most important feature among the 50 ones was the conjugation length that is measured by the number of bonds linking the farthest atom pair in a conjugation system (“Conju‐Max‐Distance”). We then calculated the performance gain after adding each of the rest 49 features using the XGBoost model. The feature providing the highest performance gain was added to the selected feature set. This procedure was repeated to select the third feature, and so on. We found that a minimum of an additional 9 features plus the “Conju‐Max‐Distance” can retain the performance of the XGBoost model: “MaxPartialCharge,” “MaxAbsPartialCharge,” “SMR_VSA10,” “VSA_EState3,” “MaxEStateIndex,” “VSA_Estate1,” “VSA_EState2,” “Wavelength (Exp nm),” “ET(30) (Solvent).”

The wavelength and solvent index are molecule‐independent features that are related to the experimental measurements. The other seven features are all “Molecular Operating Environment” (MOE) features describing the local environment of atoms in a molecule. Many of these MOE features are additive. As we would like to attribute the molecular properties to fragments containing functional groups that are familiar to chemists, we established MFF‐based MOE features (MFF‐MOE) by a simple summation to replace the atom‐based ones, including the “PEOE Charge,”^[^ ^25^ ^]^ “LogP,”^[^ ^26^ ^]^ and “MR.” “PEOE Charge” is obtained by summing up the Gasteiger charges of atoms in an MFF fragment. LogP is the logarithm of oil (octanol)–water partition coefficient of a molecule. The summation of atomic attribution of LogP to MFF can identify polar groups in the molecule. Similarly, the MR is the polarizability of the molecule determined by molar refractivity, and the summation of its atomic attribution to the MFF level can describe polarizability of a molecular fragment.

After adding a series of these new MFF‐MOE features to replace the seven atomic MOE features, we obtained a new feature matrix containing 94 features (Table S2, Supporting Information). To our surprise, after the stepwise regression, we obtained a feature set with only 6 features to give quite good performance of the XGBoost model, and only four of them are molecule‐based features, while the other two are the measurement wavelength and solvent feature. Besides the “Conju‐Max‐Distance,” “Wavelength (Exp nm),” and “ET(30) (Solvent),” the newly selected MFF‐MOE features are “PEOE‐Charge‐Max,” “LogP‐Min,” and “MR‐Max.”

### 2.4 Performance of Machine Learning Models

240 splits of training and testing sets were randomly generated to evaluate the model performances with a train‐test ratio of 85:15 (728 samples for training and 128 samples for testing). **Table** **2** listed the average MSE, MAE, R2 scores of the testing sets using the full feature matrix [856 × 696] and the selected feature matrix [856 × 6] with a bunch of different ML models (Table S3, Supporting Information). The MAE value representing the error of the prediction was as low as 0.33 in lg(TPACS) units, which corresponds to an accuracy within a factor of 2. The true‐predict scatter plots (Figure 3b,c; Section S4 and Figures S4 and S5, Supporting Information) further confirm this performance. This level of accuracy is already comparable to the accuracy of experimental measurements which are influenced by many factors, such as the measurement technique, variations of the laser pulse duration, temporal pulse shape, spatial beam profile, pulse spectrum, and pulse chirp.^[^ ^13^ ^]^ See also our analysis of differences between experimental measurements in the Supporting Information.

**Table 2:** Performance of 10 regressors on different feature matrices

Performance	[856 × 696]			[856 × 6]		
Regressor	MSE^a)^	MAE	R2	MSE	MAE	R2
AdaBoost	0.32	0.44	0.40	0.36	0.48	0.31
DNN	0.30	0.40	0.44	0.36	0.45	0.33
Decision Tree	0.41	0.48	0.22	0.37	0.46	0.29
ElasticNet	0.30	0.41	0.43	0.38	0.48	0.28
GBRT	0.22	0.34	0.59	0.23	0.37	0.56
LASSO	0.30	0.41	0.43	0.39	0.48	0.27
MLPRegressor	0.33	0.42	0.37	0.40	0.50	0.23
k‐nearest neighbor	0.39	0.47	0.25	0.42	0.49	0.19
Random Forest	0.23	0.34	0.56	0.23	0.35	0.57
XGBoost	0.22	0.33	0.59	0.21	0.35	0.58

^a)^ The accuracy of models based on mean squared error (MSE) and mean absolute error (MAE) is given in logarithmic scale. Details of these regressors are shown in Table S3 (Supporting Information). LASSO, GBRT, and XGBoost regressors are highlighted in the table.

Meanwhile, theoretical calculations of TPACS suffer from large uncertainty.^[^ ^10^, ^27^ ^]^ Even comparing the popular density functional theory (DFT) results to the benchmark calculation by coupled cluster (CC) high‐level QC method gave MAE > 0.334 in logarithm (Figure S6, Supporting Information).^[^ ^10a^ ^]^ Our simple ML model with only four molecular features thus has comparable accuracy to that of commonly used DFT methods.

### 2.5 Interpretation of the Machine‐Learning Model

**Figure 4:**
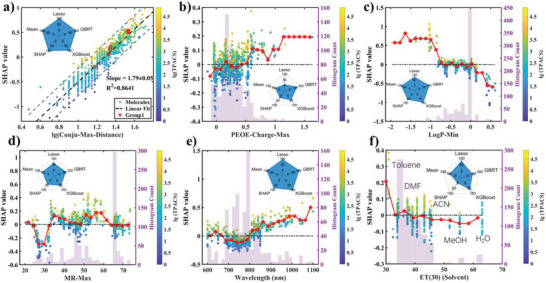
Chemical information extracted from machine learning models. a) SHAP analysis of lg(Conju‐Max‐Distance): the linear fitting gives: SHAP value = lg(Conju‐Max‐Distance)×1.79 − 2.29, and a group of structurally related compounds selected from Figure S9 (Supporting Information) is highlighted in red circle. b–d) SHAP analysis of MFF‐MOE features. e) SHAP analysis of Wavelength (Exp nm). f) SHAP analysis of ET(30). The pentagon spider insets are showing five feature importance indexes: the normalized LASSO coefficients ([856 × 696] model), the GBRT feature importance ([856 × 696] model), the XGBoost feature importance ([856 × 696] model), the sum of SHAP values based on the XGBoost model ([856 × 6] model), and the mean value of the above four indexes (Table S4, Supporting Information). The histogram of every figure shows the distribution of features.

We used the SHAP value^[^ ^28^ ^]^ as a guide to interpret the ML model (Section S5, Supporting Information). The SHAP value measures in the ML model how a specific feature contributes to the predicted TPACS of each sample. The SHAP values of different features of one sample sum up to its TPACS subtracting the mean TPACS. For a given feature, a plot of SHAP values against the feature values of different samples (SHAP plot) maps out the contribution of the feature in determining TPACS (**Figure** **4**). To analyze other feature of interest that is not included in the selected 6 features, we added the feature to the feature matrix and refit the model [856 × (6+1)] to calculate its SHAP value.

These SHAP plots allow us to test established concepts of the TPA structure–property relationship. We found that many of the concepts are consistent with the experimental statistics, but a few of them are not strongly supported.

#### 2.5.1 Conjugation Length Versus Conjugation Area Versus Molecular Weight

It has long been noticed that a larger molecule with a larger conjugated structure has a higher TPACS.^[^ ^6a^ ^]^ Noticeably, the ML model selected the conjugation length rather than the conjugation area (Conju‐Stru‐VSA) as the most critical feature. Conju‐Stru‐VSA is poorly related to the TPACS (Section S6 and Figure S7, Supporting Information).

The area of a conjugated system is also closely related to its molecular weight. Practically, in many applications,^[^ ^6a,c^ ^]^ the specific TPACS per molecular weight is of interest. It is thus important to know whether the TPACS linearly depends on the molecular weight. The plot of lg(TPACS) against the logarithm of either the whole molecular weight (Full‐Wt) or the weight of the conjugated systems in the molecule (conju‐Wt) (Figure S8, Supporting Information) showed a weak correlation.

#### 2.5.2 Is There a Quantitative Relationship between the TPACS and the Conjugated Length of a Molecule?

From the SHAP plot of the “Conju‐Max‐Distance” (Figure 4a), we observed a linear correlation between the logarithm of this feature and the SHAP value with a slope of 1.79 ± 0.05. As the SHAP value is logarithm to the TPACS, this slope corresponds to a power law of TPACS depending on the conjugation length

(1) $$\mathrm{TPACS}\propto {\left(\mathrm{Conju}-\mathrm{Max}-\mathrm{Distance}\right)}^{1.79\pm 0.05}$$

This slope of 1.79 is roughly consistent with the linear fitting of lg(TPACS) against lg(Conju‐Max‐Distance) that happens to be 1.9±0.2 with a much larger error (Figure 3d). Three groups of structurally related compounds of different conjugation lengths in the dataset (Figure S9, Supporting Information) revealed similar trend (Figure 3d). This slope is also confirmed by another data analysis method: accumulated local effects (Section S7 and Figure S10a, Supporting Information). The SHAP method thus helps us to isolate the contribution of conjugation length and extracts the first quantitative relationship between the conjugation length and the TPACS.

Beyond the statistical analyses, we also try to rationalize such a dependence based on physical models. A simple model of conjugated parallel *p*‐orbitals to form a linear *π*‐system with alternative double and single bonds showed that the lg(TPACS) is linear to the lg(Conju‐Max‐Distance) with a slope close to 1.7 in a reasonable conjugation length range (Section S8 and Figures S10b and S11, Supporting Information). More accurate time‐dependent density functional theory (TDDFT) calculations^[^ ^29^ ^]^ on a series of molecules of different conjugation lengths (Section S9, Table S5, and Figure S10c, Supporting Information) gave a lg(TPACS)‐lg(Conju‐Max‐Distance) slope of 2.4.

#### 2.5.3 The Degree of Conjugation

The “MR‐Max” uses molar refractivity to describe polarizability of a fragment. As the “MR‐Max” is correlated to the conjugation length, we used principal component analysis (Section S10, Table S6, and Figure S12, Supporting Information) to remove interference from the latter and found that the “MR‐Max” possibly manifests the degree of conjugation.

The conjugated C=C bonds, triphenylamine groups increase the SHAP value of “MR‐Max”, while a single bond connection between two aromatic rings or other substructure causing nonplanarity of the conjugation system has a negative effect (Section S11 and Figures S13c,d and S14, Supporting Information). Some heteroatoms in the conjugated system like an azo linkage between benzene rings also seem to have a negative effect.

The “MR‐Max” using polarizability as a probe is thus complementary to the conjugation length to describe the degree of conjugation of the system.

#### 2.5.4 How to Quantify the Impact of Donor and Acceptor Substitution Groups to TPACS?

Donor and acceptor substituents in the conjugated structure are critical to the TPA in a Donor‐*π*‐Acceptor design. The selected “LogP‐Min” feature can mark the existence of highly polar groups on the molecules. These groups are usually also strongly electron‐donating or electron‐withdrawing groups (Figure S13b, Supporting Information). The SHAP plot showed that the more negative this parameter (the more polar the group), the higher the TPACS, which is consistent with the push‐pull design principle (Figure 4c).

However, polarity alone cannot adequately describe the electronic property of a functional group. “PEOE‐Charge‐Max” supplements the description by identifying positively charged conjugated carbon backbone that is connected to strong electron‐withdrawing group (Figure S13a, Supporting Information), as shown by the SHAP plot that adds correction to the positively valued region (positively charged backbone) (Figure 4b).

#### 2.5.5 Is Multipolar DA_n_ or AD_n_ Structure from Branching of the Conjugated System Beneficial for TPA?

We considered multipolar DA*_n_* or AD*_n_* branched structure and quadrupolar D‐*π*‐A‐*π*‐D/A‐*π*‐D‐*π*‐A linear structure by the features of “Conju‐Branch‐Ratio” and “DAratio,” respectively. The Conju‐Branch‐Ratio is only weakly correlated to the conjugation length and adequately addresses the branching of a conjugation system in a multipolar structure (Section S12 and Figures S15a and S16, Supporting Information). However, the absolute SHAP values of the Conju‐Branch‐Ratio are mostly smaller than 0.05, indicating that it only has a minor influence on the TPACS. No higher‐order contribution of Conju‐Branch‐Ratio together with the conjugation length was observed either (Figure S15e, Supporting Information).

Similarly, the SHAP plot of the DAratio (distance between the donor and acceptor divided by the conjugation length) showed absolute values mostly smaller than 0.1 (Figure S15f, Supporting Information), suggesting a small effect. Moreover, the positive SHAP value at DAratio > 0.5 is against a beneficial effect of the quadrupolar structure, as the DAratio closer to 1 corresponds to a dipolar D–A structure rather than a quadrupolar D–A–D or A–D–A structure.

These statistical analyses of the multipolar or quadrupolar structures thus contradict the conventional wisdom about the importance of them in obtaining high TPACS. The observation of high TPACS in multipolar or quadrupolar molecules can be mainly attributed to their elongated conjugation length.

We put more analyses on other features including aliphatic chain, testing method, and solvent polarity in the Supporting Information (Section S13 and Figures S17 and S18, Supporting Information).

## 3 Experimental Validation

To test the predictive power of the ML model, 16 molecules were chosen from the chemical inventory of the lab. To evaluate the ML model's predictions, their TPACS by the Z‐scan technique which is a widely‐used standard routine for studying the nonlinear absorption coefficient^[^ ^13^, ^30^ ^]^ (see the Supporting Information for details) was measured. The selection was based on a prescreening by the ML model to make sure the chosen molecules have substantial TPACS at 800 nm. The TPACS of these 16 molecules were not reported in the literature before. Two‐photon absorption spectra of 16 molecules are shown in Figure S19 (Supporting Information). The ML model gives reasonable predictions of their TPACS as compared to the measured values at their peak wavelength (Figure S20, Supporting Information), which provides independent validation of the accuracy of our approach.

## 4 Conclusions

We obtained a simple and interpretable model to predict TPACS of different chromophores based on experimental data from literature. Despite of containing only four molecule‐based features, the model achieves a predictive accuracy comparable to both the experimental measurements and the popular density functional theory calculations. The model identifies the conjugation length as the most critical feature and gives the first quantitative relationship between the TPACS and the conjugation length. Based on this model, we also tested several popular observations in the field of two‐photon absorption research. To our surprise, we found that a widely practiced approach^[^ ^6a^ ^]^ to design DA*_n_* or AD*_n_* multipolar structure does not enhance the TPACS beyond the effect of conjugation lengthening. We envision that this simple ML model can allow fast screening of databases to accelerate the development of high‐performance organic nonlinear optical materials. An important practical requirement to be considered in such future screenings is that one‐photon absorption may hinder TPA.^[^ ^6d^ ^]^ Thus, one usually has to screen for TPA materials that have no one‐photon absorption in the desired region.^[^ ^31^ ^]^ To facilitate the use of our model and further developments, we openly provide both the database and code (see Data Availability statements). The ML TPA calculations can be conveniently performed with the MLatom package^[^ ^32^ ^]^ either locally or on the MLatom@XACS cloud computing service.^[^ ^33^ ^]^

## Conflict of Interest

The authors declare no conflict of interest.

## Supporting information

Supporting Information

## Data Availability

The data that support the findings of this study are openly available in figshare at https://doi.org/10.6084/m9.figshare.c.6264228.v2, reference number 6264228. All implementations reported in this work are openly available on Github at https://github.com/Wang‐Group/TPAML. Predictions of ML‐TPA cross section can be done with open source, free package MLatom at http://mlatom.com. In addition, calculations can be performed online using the MLatom@XACS (Xiamen Atomistic Computing Suite) cloud computing service at http://XACScloud.com.
