# Supplementary material for: Interpretable Machine Learning of Two‐Photon Absorption
Source: Adv Sci (Weinh). 2023 Jan 19;10(8):2204902. doi: 10.1002/advs.202204902 (PMC10015897; doi:10.1002/advs.202204902)
Supplement: Supplementary file 1 — Supporting Information [file ADVS-10-2204902-s001.pdf]

Supplementary information

Section 1. The dataset from literature reports

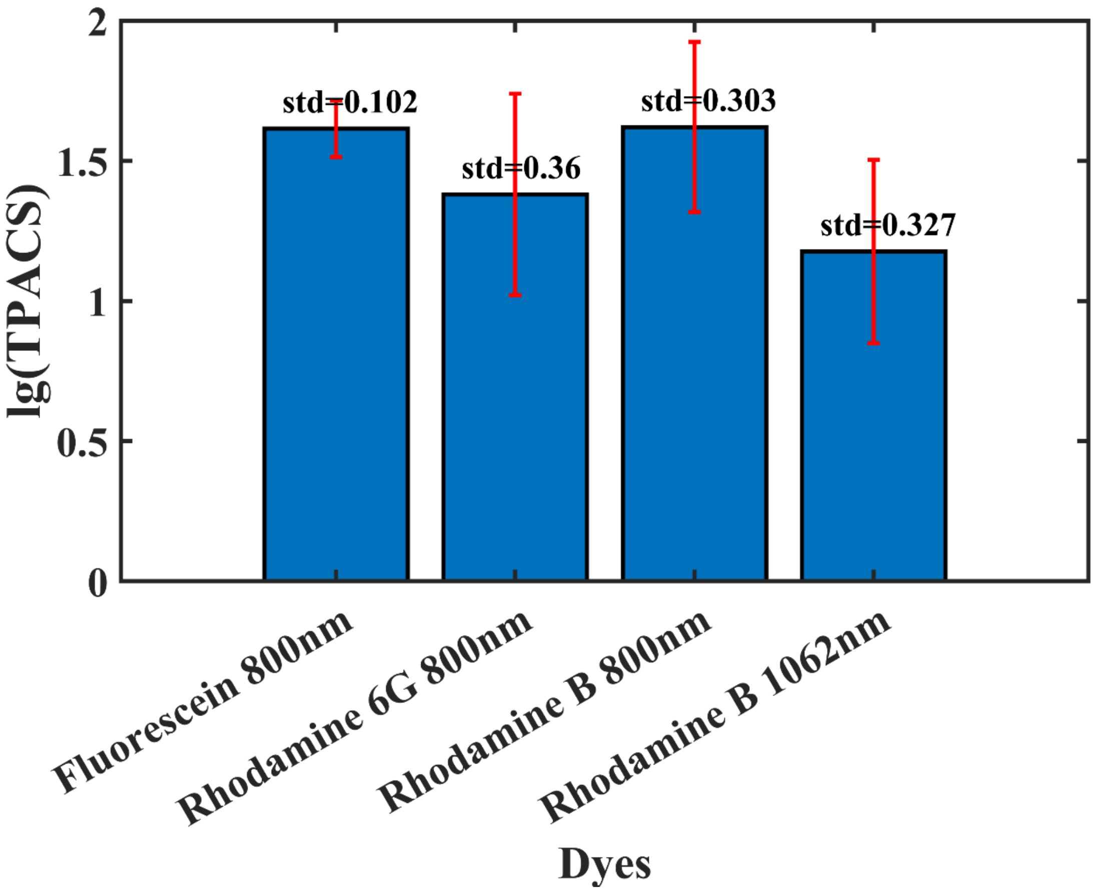

Figure S1. TPACS values of some common commercially available dyes from different sources.[1] The standard deviation was noted above the bar. These discrepancies are possibly introduced because the TPACS measurements depend on the femtosecond laser pulse, which may vary from instrument to instrument.

Section 2. Descriptions of all the features

2.1 MFF Features

The concept of Molecular Fragment Fingerprint[2] was inspired by ECFP provided by Deepchem[3], while the hash function of the features was bypassed and the interpretable

molecular fragments were generated. An occurrence frequency threshold was set to drop features that appeared too few times. Thus, useful substructures of a molecule were extracted. There are 564 MFF features included in our dataset.

2.2 RDKit Features

We use all the available descriptors given by RDKit.[4] There are 107 RDKit features included.

2.3 Conjugation Features

2.3.1 'Num-Conju-Stru'

The number of conjugation structures in one molecule. Each conjugation structure contains more than one atoms in the π-system.

$$F_i = N_{conju-stru,i}$$

F_i represents this feature for a particular molecule, where the subscript i is the index of the molecule in the dataset.

2.3.2 'Conju-Num-Atom-All'

The maximum number of atoms of all conjugation structures in one molecule. This descriptor was not included in the first round of machine learning.

$$F_i = \max\{N_{atoms\ of\ conju-stru,i,j}\}$$

The subscript j is the index of conjugation structure in the ith molecule.

2.3.3 'Conju-Num-Atom-Ratio'

The number of atoms in all conjugation structures divided by the number of atoms in one molecule.

$$F_i = \frac{\sum_{j=1}^{N_{conju-stru,i}} N_{atoms,i,j}}{N_{all\ atoms,i}}$$

$N_{all\ atoms,i}$ is the number of atoms in the ith molecule.

2.3.4 'Conju-Num-Atom-Individual'

The number of atoms in conjugation structures in one molecule. If the molecule contains

several independent conjugated sub-structures, the number of atoms of those independent ones should be considered as well.

$$F_i = \text{sum}\{N_{atoms\ of\ conju-stru,i,j}\}$$

2.3.5 'Conju-Wt-Ratio'

The sum of weight of all conjugation structures divided by the molecular weight.

$$F_i = \frac{\sum_{j=1}^{N_{conju-stru,i}} Wt_{conju-stru,i,j}}{Wt_{mol,i}}$$

$Wt_{mol,i}$ is the molecular weight of the ith molecule.

2.3.6 'Conju-Wt-Part'

The maximum weight of all conjugation structures in one molecule. The weight of conjugation structure is calculated by adding up the atomic weight of atoms it contains.

$$Wt_{conju-stru,i,j} = \sum_{k=1}^{N_{atoms,i,j}} Wt_{atom,i,j,k}$$

$N_{atoms,i,j}$ stands for the number of atoms in the jth conjugation structure of the ith molecule. The subscript k means the index of atom in the jth conjugation structure of the ith molecule.

$$F_i = \max\{Wt_{conju-stru,i,j}\}$$

2.3.7 'Conju-Wt-Ave'

The maximum atomically averaged weight of all conjugation structures in one molecule. The atomically averaged weight of conjugation structure is calculated by dividing the weight of conjugation structure by the number of atoms it contains.

$$Wt_{conju-stru\ atom,i,j} = \frac{1}{N_{atoms,i,j}} * Wt_{conju-stru,i,j}$$

$$F_i = \max\{Wt_{conju-stru\ atom,i,j}\}$$

2.3.8 'Conju-Max-Distance'

The maximum conjugated length of all conjugation structures in one molecule. First, the distance matrix was obtained by RDKit by "Chem.rdmolops.GetDistanceMatrix(mol)". Then, the conjugated sub-structures were selected and the maximum of its distance matrix was calculated.

$$F_i = \max\{d_{i,j,p,q}\}$$

$d_{i,j,p,q}$ is the shortest distance between the p th and the q th atoms in the j th conjugation substructure of the ith molecule.

2.3.9 'Conju-Branching-Index '

The maximum branching index of all conjugation structures in one molecule. The branching index of one conjugation structure is defined as the number of atoms it contains dividing by half of the sum of degrees of atoms.

$$I_{branching,i,j} = \frac{N_{atoms,i,j}}{\frac{1}{2} * \sum_{k=1}^{N_{atoms,i,j}} D_{i,j,k}}$$

$D_{i,j,k}$ stands for the degree of the kth atom in the jth conjugation structure of the ith molecule. The degree of one atom is equal to the number of its neighbor atoms.

$$F_i = \max\{I_{branching,i,j}\}$$

2.3.10 'Conju-Branch-Ratio'

We count the number of possible conjugated paths of the maximum conjugated length. If this number is beyond 1, branching structure must exist in the molecule. We then try to determine if the branching happens at the middle of the conjugated system or at the rim of the system.

First, we find two conjugated paths of the maximum conjugated length, AB and AB', sharing a common end atom A. We then find the other end atoms of the two paths, B and B' respectively. We calculated the distance between B and B' L_b.

The 'Conju-Branch-Ratio' equals to L_b divided by the 'Conju-Max-Distance' L_{conj}.

$$F_i = \frac{L_b}{L_{conj}}$$

As a result, a 'Conju-Branch-Ratio' close to 1 corresponds to a branching in the middle of the molecule.

2.3.11 'Full-Mol-Wiener-Index'

Wiener Index of the whole molecule. Wiener Index is calculated by the formula below:

$$I_{Wiener} = \frac{1}{n * (n-1)} * \sum_{p=1}^{n} \sum_{q=1}^{n} d_{p,q}$$

n is the number of nodes in the graph, and $d_{p,q}$ is the shortest distance between the pth and the qth nodes.

The Wiener Index of a molecule is calculated by the formula below:

$$F_i = I_{Wiener,i} = \frac{1}{N_{all\ atoms,i} * (N_{all\ atoms,i} - 1)} \sum_{p=1}^{N_{all\ atoms,i}} \sum_{q=1}^{N_{all\ atoms,i}} d_{i,p,q}$$

$d_{i,p,q}$ is the shortest distance between the pth and the qth atoms in the ith molecule.

2.3.12 'Conju-Stru-Wiener-Index'

Wiener Index of the conjugation structure with the maximum size in one molecule.

$$F_i = I_{Wiener,i,J} = \frac{1}{N_{atoms,i,J} * (N_{atoms,i,J} - 1)} \sum_{p=1}^{N_{atoms,i,J}} \sum_{q=1}^{N_{atoms,i,J}} d_{i,J,p,q}$$

The subscript J is the index of the conjugation structure containing maximum number of atoms in the ith molecule.

2.3.13 'Conju-Stru-VSA'

The approximate surface area of all conjugation structures in one molecule. If we take the shape of each atom to be a sphere with radius equal to the van der Waals radius, we obtain the van der Waals surface area (VSA) for each atom. The sum of the VSA of each atom gives the molecular VSA.

Let us consider a molecule of n atoms. Each atom has a van der Waals radius of R_i, and let B_i denotes the set of all atoms bonded to the atom i. Distance between sphere A and B is d_i. b_{ij} is a pre-defined reference bond length between atom i and atom j. We will neglect the effect of atoms not related by a bond to the atom i and define the VSA for atom i, denoted by V_i, to be:

$$V_i = 4\pi {R_i}^2 - \pi R_i \sum_{j=1}^{B_i} \frac{R_j - (R_i - d_{ij})^2}{d_{ij}}$$

$$d_{ij} = \min\{\max\{|R_i - R_j|, b_{ij}\} R_i + R_j\}$$

$$F_i = \max\{ \sum_{p}^{N_{atoms,i,j}} V_i\}$$

2.3.14 'Conju-Branch-Num'

Half of the number of paths with the maximum distance ('Conju-Max-Distance') in the conjugated structure.

2.3.15 'Conju-sp2N-Num'

The number of N atom with sp2 hybridization in all conjugation structures in one molecule.

2.3.16 'Apparent-Conju-Electron-Count'

The maximum apparent electron counts of all conjugation structures in one molecule. The apparent electron count is calculated by adding up all the electron contributed by the atoms to the conjugation structure.

$$N_{app-elec,i,j} = \sum_{k=1}^{N_{atoms,i,j}} N_{atom-contribute,i,j,k}$$

$N_{atom-contribute,i,j,k}$ stands for the number of electrons the kth atom 'apparently' contributes to the jth conjugation structure in the ith molecule as shown in the table below:

Atom type	$N_{atom-contribute}$
C atom (sp^2 or sp hybridization)	1
N atom (sp^2 hybridization)	2
N atom (sp hybridization)	1
O atom (sp^3 hybridization)	2
O atom (sp^2 hybridization)	1
Halogen atoms (F, Cl, Br, I)	2
Other heavy atoms (S, P)	2

This feature is calculated by the formula below:

$$F_i = \max\{N_{app-elec,i,j}\}$$

2.3.17 'Conju-Elec-Influence'

The electron-influence is calculated by summing up the product of the atom's number of neighboring non-H atoms and the number of electrons it contributes to the conjugation structure.

$$I_{elec-influ,i,j} = \sum_{k=1}^{N_{atoms,i,j}} D_{i,j,k} * N_{atom-contribute,i,j,k}$$

$D_{i,j,k}$ stands for the number of neighboring non-H atoms of the kth atom in the jth conjugation structure of the ith molecule.

The definition of $N_{atom-contribute,i,j,k}$ is already mentioned before.

$$F_i = \max\{I_{elec-influ,i,j}\}$$

2.3.18 'Conju-Elec-Influence-Ave'

The atomic normalization of electron-influence of the conjugation structures:

$$I_{elec-atom-influ,i,j} = \frac{1}{N_{atoms,i,j}} \sum_{k=1}^{N_{atoms,i,j}} D_{i,j,k} * N_{atom-contribute,i,j,k}$$

$$F_i = \max\{I_{elec-atom-influ,i,j}\}$$

2.3.19 'Conju-Elec-Distance-Coef'

The maximum of electronic distance coefficient of all conjugation structures in one molecule. The electronic distance coefficient is calculated by the formula below:

$$I_{elec-distance-coef,i,j} = \sum_{p=1}^{N_{atoms,i,j}} \sum_{q=p+1}^{N_{atoms,i,j}} d_{i,j,p,q} * N_{atom-contribute,i,j,p} * N_{atom-contribute,i,j,q}$$

Note that q starts from $p+1$ so that all atom pairs are calculated only once.

$d_{i,j,p,q}$ is the shortest distance between the pth and the qth atoms in the jth conjugation substructure of the ith molecule.

The natural logarithm of result is taken as the feature:

$$F_i = \max\{\log(I_{elec-distance-coef,i,j})\}$$

2.3.20 'Conju-Elec-Distance-Coef-Norm'

The maximum of normalized electronic distance coefficient of all conjugation structures in one molecule. The normalization is done by dividing the electronic distance coefficient by the number of atom pairs.

$$I_{norm-elec-distance-coef,i,j} = \frac{1}{\left(N_{atoms,i,j} - 1\right) * (N_{atoms,i,j} - 2)} I_{elec-distance-coef,i,j}$$

This value is relatively small so that it is not needed to take the form of logarithm:

$$F_i = \max\{I_{norm-elec-distance-coef,i,j}\}$$

2.3.21 'Conju-MultiElec-Distance-Coef'

The maximum of multi-electronic distance coefficient of all conjugation structures in one molecule. Its calculation is similar to that of the 'Conju-Elec-Distance-Coef', but the number of electrons contributing to the conjugation structure is subtracted by 1:

$$I_{multi-elec-distance-coef,i,j} = \sum_{p=1}^{N_{atoms,i,j}} \sum_{q=p+1}^{N_{atoms,i,j}} d_{i,j,p,q} * \left(N_{atom-contribute,i,j,p} - 1\right) * (N_{atom-contribute,i,j,q} - 1)$$

$$F_i = \max\{I_{multi-elec-distance-coef,i,j}\}$$

2.3.22 'Conju-MultiElec-Distance-Coef-Norm'

The maximum of normalized multi-electronic distance coefficient of all conjugation structures in one molecule. The normalization is also done by dividing the multi-electronic distance coefficient by the number of atom pairs.

$$I_{norm-multi-elec-distance-coef,i,j} = \frac{1}{\left(N_{atoms,i,j} - 1\right) * (N_{atoms,i,j} - 2)} I_{multi-elec-distance-coef,i,j}$$

$$F_i = \max\{I_{norm-multi-elec-distance-coef,i,j}\}$$

2.4 MFF-based MOE features

Five categories of properties could be calculated in our featurization algorithm (MFF-MOE): 'Apperant-Elec-Count', 'PEOE-Charge', 'EState-Indice', 'LogP', 'MR'. In this section, only the longest conjugated sub-structure is considered. The value of the properties of MFF is obtained by the summation of values of its containing atoms.

Taking "PEOE Charge" as an example, we sum up the Gasteiger atomic charges of atoms in an MFF fragment. The maximum and minimum of these summed PEOE charges of all fragments were then extracted as two molecular features: "PEOE-Charge-Max" and "PEOE-Charge-Min".

LogP is the logarithm of oil (octanol)–water partition coefficient of a molecule. The atomic attribution of LogP effectively explores the local polarity of a molecule. The summation of atomic LogP to MFF can identify polar groups in the molecule. Similarly, the MR is the polarizability of the molecule determined by molar refractivity. The atomic attribution of the MR

highlights the polarizability of each atom in a molecule, while its summation to the MFF shows the polarizability of a conjugated fragment.

The 'Apperant-Elec-Count' is already defined in the previous section. The atomic PEOE charge, EState indice, contribution of LogP, and contribution of MR are calculated by the RDKit toolkit. The "Estate" parameters in our models are NOT electronic states numbers.

The following features have **'P_x'** in the names. The **'P_x'** stands for names of different properties as shown in the table below.

P_x	property
x=1	Apperant-Elec-Count
x=2	PEOE-Charge
x=3	EState
x=4	LogP
x=5	MR

'$P_{x,i,k}$' means the xth atomic property of the kth atom in the ith molecule.

2.4.1 'P_x -Sum'

The sum of atomic properties of atoms in the conjugation structure. If multiple conjugation structures are present in one molecule, then the value of the one with the maximum number of atoms will be used (similarly hereinafter).

$$F_{x,i} = \sum_{k}^{N_{atoms,i,j}} P_{x,i,j,k}$$

2.4.2 'P_x-Ave'

The atomic averaged value of atomic properties of atoms in the conjugation structure.

$$F_{x,i} = \frac{1}{N_{atoms,i,j}} \sum_{k}^{N_{atoms,i,j}} P_{x,i,j,k}$$

2.4.3 'P_x-Max'

The maximum value of properties of single atoms and all possible MFF fragments in the conjugation structure. The sum of atomic properties in a MFF fragment is calculated first as fragment property. Then the maximum value is picked out from all the single atom properties together with all fragment properties.

$$P_{x,i,j,m} = \sum_{k}^{N_{atoms,i,j,m}} P_{x,i,j,k}$$

The fragment properties will be noted as '$P_{x,i,j,m}$' which means the property of the mth fragment in the jth conjugation structure of the ith molecule. $N_{atoms,i,m}$ represents the atom index of the mth fragment in the jth conjugation structure of the ith molecule.

$$F_{x,i} = \max(P_{x,i,j,k}(k = 1, 2, \ldots, N_{atoms,i,j}), P_{x,i,j,m}\ (m = 1, 2, \ldots, N_{frags,i,j}))$$

$N_{frags,i,j}$ means the number of fragments in the jth conjugation structure of the ith molecule.

2.4.4 'P_x-Min'

The minimum value of properties of single atoms and all possible MFF fragments in the conjugation structure.

$$F_{x,i} = \min(P_{x,i,j,k}(k = 1, 2, \ldots, N_{atoms,i,j}), P_{x,i,j,m}\ (m = 1, 2, \ldots, N_{frags,i,j}))$$

2.4.5 'P_x-Delta'

The difference between the maximum and minimum properties of single atoms and all possible MFF fragments in the conjugation structure.

$$F_{x,i} = \max\left(P_{x,i,j,k}\left(k = 1, 2, \ldots, N_{atoms,i,j}\right), P_{x,i,j,m}\ \left(m = 1, 2, \ldots, N_{frags,i,j}\right)\right) - \min\left(P_{x,i,j,k}\left(k = 1, 2, \ldots, N_{atoms,i,j}\right), P_{x,i,j,m}\ \left(m = 1, 2, \ldots, N_{frags,i,j}\right)\right)$$

2.4.6 'P_x-Weighted'

The weighted atomic properties of all atoms in the conjugation structures. The weighted property is calculated by summing up the product of the atom's number of neighboring non-H atoms and the respective atomic property.

$$F_{x,i} = \sum_{k=1}^{N_{atoms,i,j}} D_{i,j,k} * P_{x,i,j,k}$$

$D_{i,j,k}$ is the number of neighbor atoms of the kth atom of the jth conjugation structure in the ith molecule.

2.4.7 'P_x-Weighted-Ave'

The atom averaged weighted atomic properties of all atoms in the conjugation structures.

$$F_{x,i} = \frac{1}{N_{atoms,i,j}} \sum_{k=1}^{N_{atoms,i,j}} D_{i,j,k} * P_{x,i,j,k}$$

2.4.8 'P$_x$-PositiveDisCoef'

The one order distance coefficient of the atomic properties of all atoms in the conjugation structures. The one order distance coefficient is calculated by the formula below:

$$F_{x,i} = \sum_{p=1}^{N_{atoms,i,j}} \sum_{q=p+1}^{N_{atoms,i,j}} d_{i,j,p,q} * P_{x,i,j,p} * P_{x,i,j,q}$$

It is noted that q starts from $p + 1$ so that all atom pairs are calculated only once.

2.4.9 'P$_x$-PositiveDisCoef-PairMean'

The averaged one order distance coefficient of the atomic properties of all atoms in the conjugation structures.

$$F_{x,i} = \frac{1}{\left(N_{atoms,i,j} - 1\right) * (N_{atoms,i,j} - 2)} \sum_{p=1}^{N_{atoms,i,j}} \sum_{q=p+1}^{N_{atoms,i,j}} d_{i,j,p,q} * P_{x,i,j,p} * P_{x,i,j,q} \ (\max N_{atoms,i,j})$$

2.4.10 'P$_x$-NegativeDisCoef'

The negative one order distance coefficient of the atomic properties of all atoms in the conjugation structures. The negative one order distance coefficient is calculated by the formula below:

$$F_{x,i} = \sum_{p=1}^{N_{atoms,i,j}} \sum_{q=p+1}^{N_{atoms,i,j}} \frac{P_{x,i,j,p} * P_{x,i,j,q}}{d_{i,j,p,q}}$$

2.4.11 'P$_x$-NegativeDisCoef-PairMean'

The averaged negative one order distance coefficient of the atomic properties of all atoms in the conjugation structures.

$$F_{x,i} = \frac{1}{\left(N_{atoms,i,j} - 1\right) * (N_{atoms,i,j} - 2)} \sum_{p=1}^{N_{atoms,i,j}} \sum_{q=p+1}^{N_{atoms,i,j}} \frac{P_{x,i,j,p} * P_{x,i,j,q}}{d_{i,j,p,q}}$$

2.4.12 'P$_x$-GradSum'

The sum of gradient of the atomic properties of all atoms in the conjugation structures.

$$F_{x,i} = \sum_{p=1}^{N_{atoms,i,j}} \sum_{q=p+1}^{N_{atoms,i,j}} \frac{\left|P_{x,i,j,p} - P_{x,i,j,q}\right|}{d_{i,j,p,q}}$$

2.4.13 'P$_x$-GradSum-PairMean'

The averaged gradient of the atomic properties of all atoms in the conjugation structures.

$$F_{x,i} = \frac{1}{\left(N_{atoms,i,j} - 1\right) * \left(N_{atoms,i,j} - 2\right)} \sum_{p=1}^{N_{atoms,i,j}} \sum_{q=p+1}^{N_{atoms,i,j}} \frac{\left|P_{x,i,j,p} - P_{x,i,j,q}\right|}{d_{i,j,p,q}}$$

2.4.14 'P$_x$-LaplaceSum'

The sum of Laplace gradient of the atomic properties of all atoms in the conjugation structures.

$$F_{x,i} = \sum_{p=1}^{N_{atoms,i,j}} \sum_{q=p+1}^{N_{atoms,i,j}} \frac{\left|P_{x,i,j,p} - P_{x,i,j,q}\right|}{d_{i,j,p,q}^2}$$

2.4.15 'P$_x$-Laplace-PairMean'

The averaged laplace gradient of the atomic properties of all atoms in the conjugation structures.

$$F_{x,i} = \frac{1}{\left(N_{atoms,i,j} - 1\right) * \left(N_{atoms,i,j} - 2\right)} \sum_{p=1}^{N_{atoms,i,j}} \sum_{q=p+1}^{N_{atoms,i,j}} \frac{\left|P_{x,i,j,p} - P_{x,i,j,q}\right|}{d_{i,j,p,q}^2}$$

2.4.16 'P$_x$-MaxMinDisRatio'

The ratio between the distance of the single atoms or fragments with the maximum property and the minimum property over the maximum distance of conjugation structure.

$$F_i = \frac{\max\{d_{i,j,p',q'}\}}{\max\{d_{i,j,p,q}\}}$$

p', q' is the atomic index of the single atom or the fragment with maximum and minimum properties. $d_{i,j,p',q'}$ stands for the longest distance of two atoms in the fragments with maximum and minimum properties.

2.5 other features

2.5.1 'DAratio'

The ratio between the distance of the single atoms with the maximum PEOE charge and the minimum PEOE charge and the maximum distance of conjugation structure.

$$F_i = \frac{\max\{d_{i,j,p',q'}\}}{\max\{d_{i,j,p,q}\}} (\max N_{atoms,i,j})$$

p', q' means the atomic index of the single atom with maximum and minimum PEOE charge. $d_{i,j,p',q'}$ stands for the longest distance of two atoms in the atoms with maximum and minimum PEOE charge.

Section 3. Feature importance

For LASSO, the magnitude of coefficients can be used as importance of the corresponding feature. For the GBRT and XGB Regressor, SHAP[5], a Python library to calculate Shapley values, was implemented to generate more interpretable feature importance. As for each regressor, 240 rounds of cross validation with randomly generated training-testing splits were carried out, and the feature importance of each round was collected, multiplied by the R^2 score of respective round, and then added up as weighted accumulated feature importance.

Then the accumulated feature importance of each regressor was sorted and ranked. The normalized ranking score of each feature on the three lists were then multiplied by the regressor's respective weight and added up to give a combined feature importance index. The weight coefficient for LASSO, GBRT and XGBoost Regressor were 0.15, 0.35 and 0.5, based on their different performances.

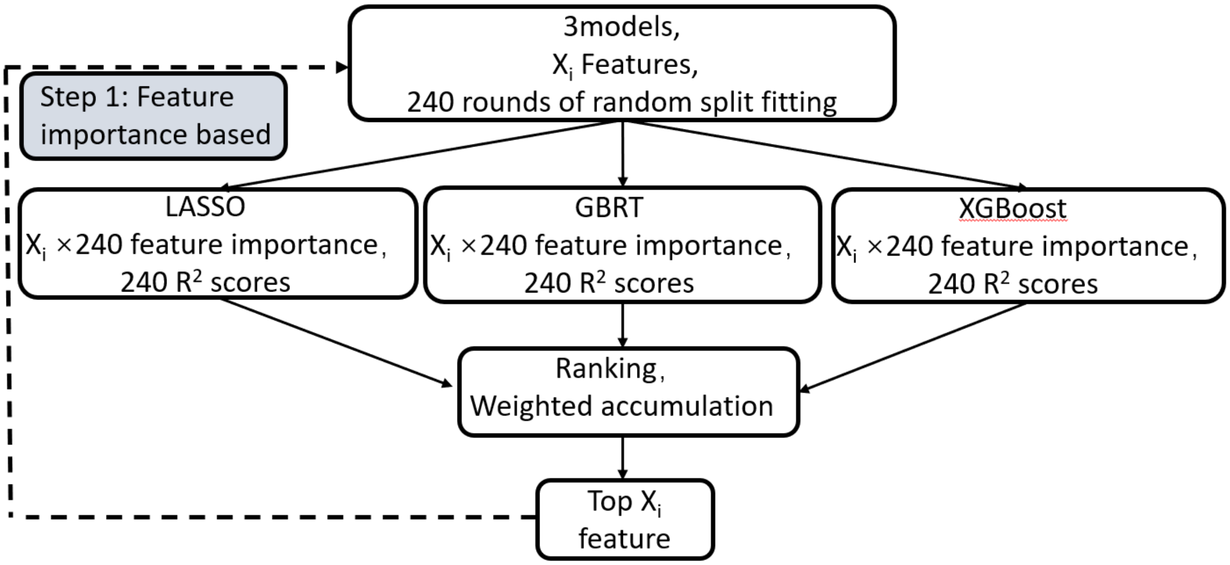

Figure S2. Feature importance-based feature selection

Table S1. Ranking and score details based on 856×50 feature matrix.

Feature	Lasso Rank	GBRT Rank	XGBoost Rank	Stepwise-regression rank
Conju-Max-Distance	6	3	2	1
Wavelength (Exp nm)	8	1	3	4
Conju-Stru-Wiener-Index	49	2	1	26
VSA_EState2	2	7	8	7
SMR_VSA6	9	4	9	19
Kappa3	26	12	5	31
Conju-Elec-Distance-Coef	1	9	12	35
SMR_VSA10	41	5	7	3
Chi2n	46	10	4	48
C=Cc1ccc(N)cc1	47	8	6	27
C=Cc	7	15	14	11
MinPartialCharge	17	21	10	17
cccc(c)C	29	17	11	12
EState_VSA10	5	18	16	14

SMR_VSA3	15	11	19	34
Conju-Elec-Influence	34	14	15	37
VSA_EState1	33	16	18	10
VSA_EState3	20	24	17	5
Conju-Wt-Ratio	13	19	21	32
Conju-Elec-Distance-Coef-Norm	39	6	24	41
Kappa1	45	25	13	30
SlogP_VSA8	43	13	25	23
PEOE_VSA8	38	20	23	47
MaxEStateIndex	40	23	22	6
HallKierAlpha	44	26	20	40
ccc	30	30	26	39
ccc(cc)-c(c)c	3	39	27	20
MaxPartialCharge	10	29	32	2
SMR_VSA9	22	36	28	29
EState_VSA6	21	34	33	28
Conju-Part-Wt	36	22	38	24
EState_VSA2	14	48	29	25
ccc(cc)N(C)C	4	27	42	42
MolLogP	18	28	39	44
SlogP_VSA5	25	33	36	45
SMR_VSA7	42	37	31	50
FractionCSP3	23	40	35	18
SlogP_VSA1	37	38	34	15
ET(30) (Solvent)	32	49	30	8
Conju-Branch-Ratio	16	41	40	21
PEOE_VSA9	28	35	45	36
MaxAbsPartialCharge	12	32	50	9
cc(c)-c	24	46	41	13
ccccc	35	50	37	16
MinAbsEStateIndex	27	45	43	49
BertzCT	50	31	47	22
Full-Mol-Wiener-Index	19	43	48	46
VSA_EState10	11	44	49	38
Conju-Elec-Influence-Ave	31	47	44	33
PEOE_VSA7	48	42	46	43

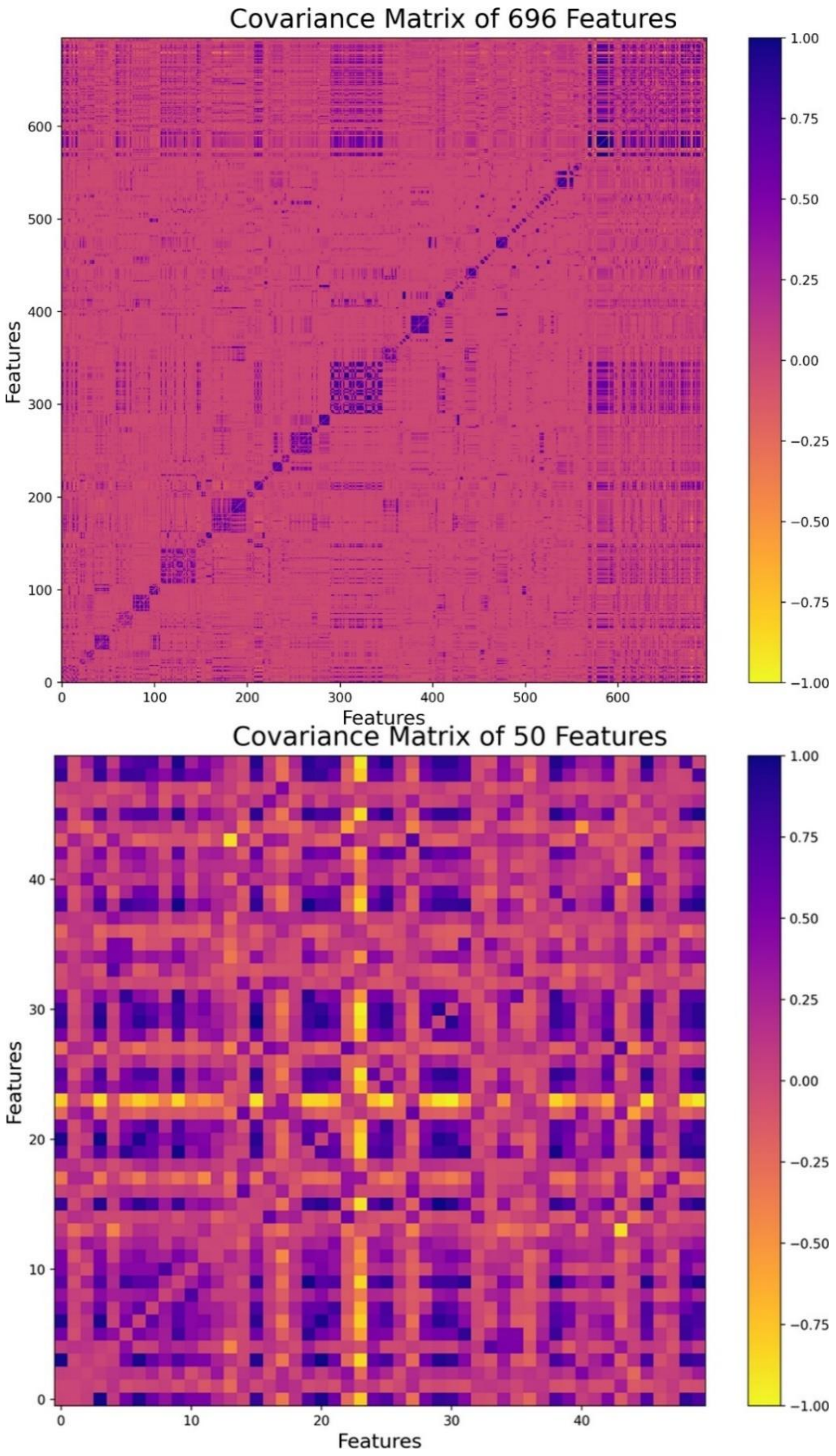

Figure S3. Covariance matrices of 696 features and 50 features. The values are the covariance between two features divided by the product of standard deviations of the two features.

Table S2. The 94 features used in the second round of stepwise regression.

Name	Number	Description
MFF-MOE	80	Describing molecular structure and functional groups.
Conjugation descriptors	12	Describing the properties of conjugation structure.
Solvent descriptor	1	ET(30) to describing the polarity of solvents.
Wavelength	1	Experimental TPA wavelength.

Section 4. Machine-Learning models and performances

Table S3. ML model parameters used by 10 regressors.[a]

**Regressor**	**Dependent package name**	**Parameters**
AdaBoost	sklearn.ensemble.AdaBoostRegressor	DecisionTreeRegressor(max_depth=4), n_estimators=300
DNN	tensorflow.keras	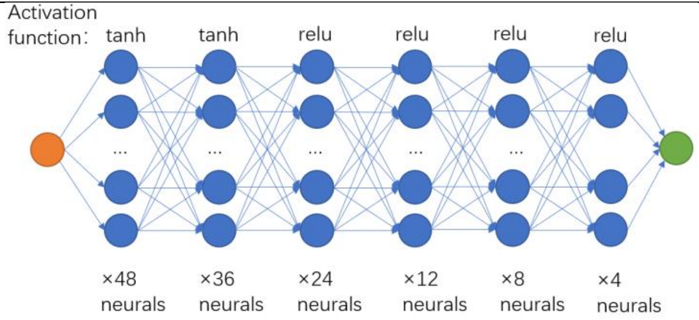
Decision Tree	sklearn.tree. DecisionTreeRegressor	max_depth=5
ElasticNet	sklearn.linear_model.ElasticNet	alpha=6.0, l1_ratio=0.5

GBRT	sklearn.ensemble.GradientBoostingRegressor	n_estimators=200, verbose=0, loss='ls', validation_fraction=0.15, n_iter_no_change=50, tol=0.00025, subsample=0.5,warm_start=False, learning_rate=0.045, min_impurity_decrease=0.003972950783280587,max_depth=9, max_features=0.2913910225812219, max_leaf_nodes=14
Lasso	sklearn.linear_model.Lasso	alpha=3.0, max_iter=8000, tol=0.005, selection='random', precompute=False
MLPRegressor	sklearn.neural_network.MLPRegressor	hidden_layer_sizes=(30,15),learning_rate_init=0.1,early_stopping=True,random_state=0
k-nearest neighbor	sklearn.neighbors.KNeighborsRegressor	n_neighbors=4,leaf_size=50
Random Forest	sklearn.ensemble.RandomForestRegressor	random_state=1,n_estimators=500
XGBoost	xgboost.sklearn.XGBRegressor	n_estimators=150, learning_rate=0.025, max_depth=13, verbosity=0, booster='gbtree', reg_alpha=np.exp(-6.788644799030888), reg_lambda=np.exp(-7.450413274554533), gamma=np.exp(-5.374463422208394), subsample=0.5, objective= 'reg:squarederror'

[a] The parameters of LASSO, GBRT and XGBoost models were obtained by Beyesian optimization method. Other parameters not mentioned were used as default.

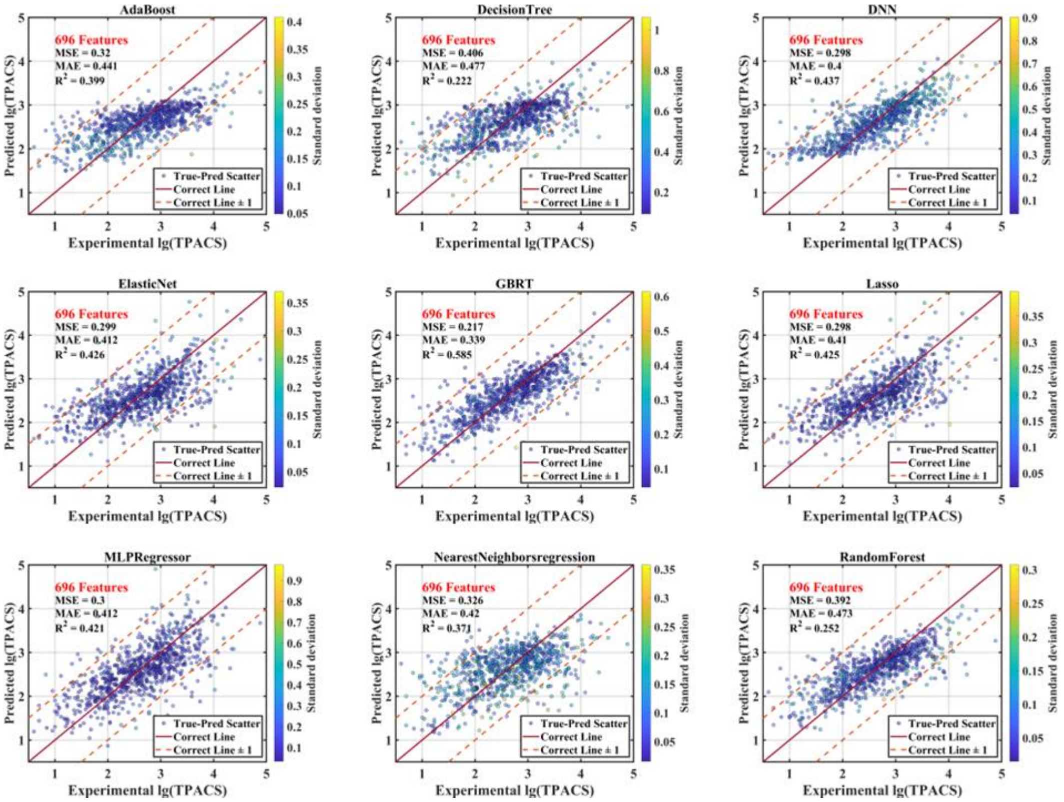

Figure S4. True-Predict Scatter Plot using the [856◊696] feature matrix with 9 regressors.

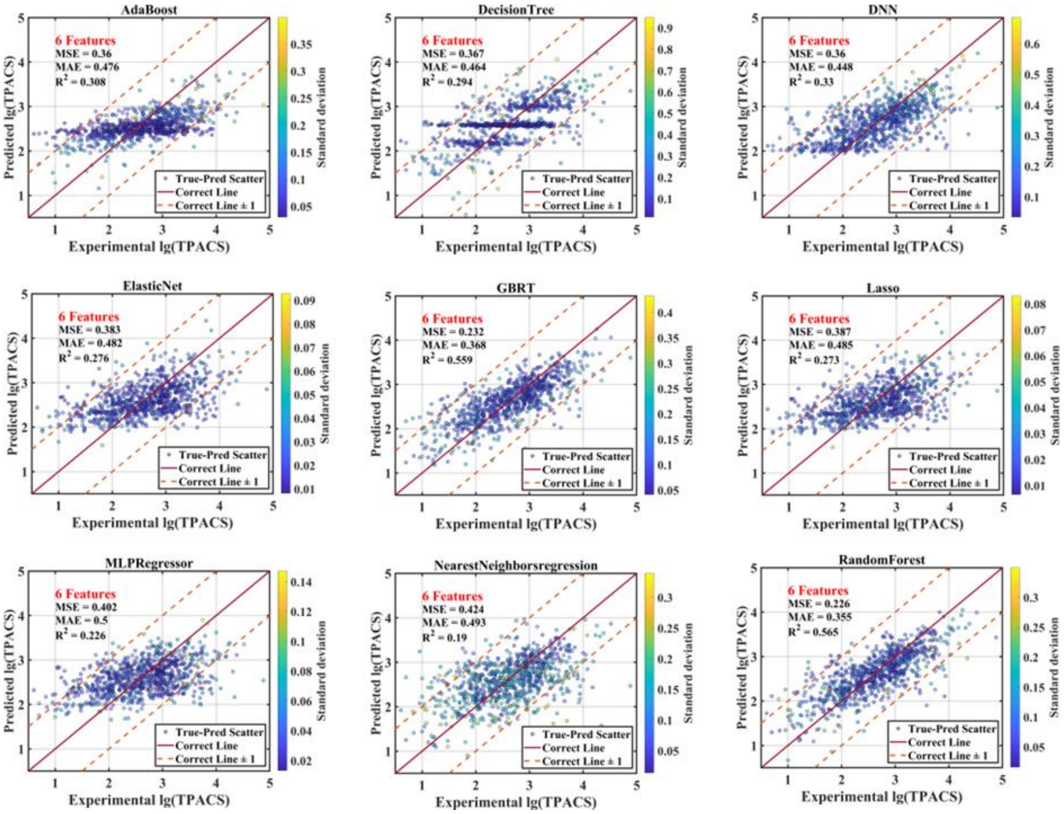

Figure S5. True-Predict Scatter Plot using the [856◊6] feature matrix with 9 regressors.

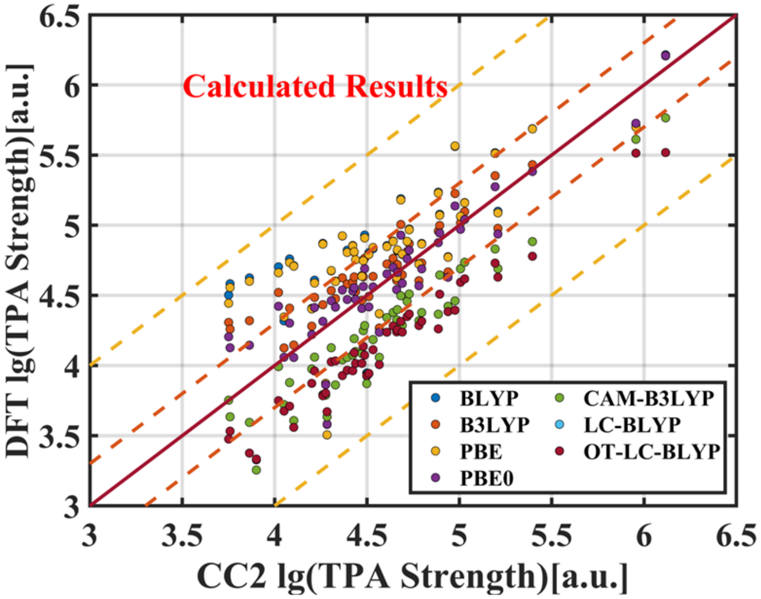

DFT	MSE	MAE	R2
BLYP	0.169	0.351	0.561
B3LYP	0.073	0.210	0.723
PBE	0.162	0.346	0.571
PBE0	0.055	0.170	0.776
CAM-B3LYP	0.144	0.349	0.902
LC-BLYP	0.202	0.437	0.955
OT-LC-BLYP	0.202	0.437	0.955

Figure S6. Calculated TPACS of 48 molecules mentioned by Robert Zaleśny[6] based on 7 different DFT settings. The results were shown in base 10 logarithm. The performance was listed on the right. The red dashed lines and yellow dashed lines stand for lines of ±0.3 and ±1.

Section 5. The SHAP value

The sum of SHAP values of different features of a given sample equals to the deviation of the predicted TPACS of this sample from the mean TPACS value of the whole training samples. In linear models like LASSO, the feature contribution is proportional to its value, while the relationships between feature contributions and feature values are mostly nonlinear in more complex models like the tree models, which is called the heterogeneity of features. These SHAP plots provide important information for interpreting machine learning results.

Here we used the XGBoost models [856 × 6] in 240 CV runs together with SHAP to generate the plots of feature contributions. The sum of the absolute SHAP values of all the samples for each feature (based on [856 × 6] feature matrix) serves as an additional feature importance measurement (Table S4).

Table S4. Feature importance indexes of the six selected features.

Feature names	LASSO score	GBRT score (based on [856 × 696] feature matrix)	XGBoost score (based on [856 × 696] feature matrix)	SHAP score (XGBoost based on [856 × 6] feature matrix)	Mean score	MSE decrease in stepwise regression
Conju-Max-Distance	96.96774	94.94624	96.96774	100	97.22043	0.288
PEOE-Charge-Max	6	60.58065	45.41935	20	33	0.0688
LogP-Min	76.75269	96.96774	94.94624	60	82.16667	0.0762
Wavelength (Exp nm)	90.90323	100	100	80	92.72581	0.0343
MR-Max	57.54839	58.55914	54.51613	40	52.65591	0.0108
ET(30) (Solvent)	74.73118	36.32258	58.55914	0	42.40323	0.006

Section 6. Comparison between conjugated length and conjugated area

The conjugated length and the conjugated area are correlated and are thus both correlated with the TPACS. We carefully compared these two features to show that the conjugated length is a better descriptor to choose.

From the scatter plot of Conju-Max-Distance (conjugated length) vs. Conju-Stru-VSA (conjugated area) color-coded by lg(TPACS) values, we can see that the conjugated length and the conjugated area are highly correlated, as expected. (Figure S7a). However, it is clear from the figure that at the same conjugated area, the TPACS positively correlated with the Conju-Max-Distance (horizontal line from left to right). In comparison, at the same conjugated length, the TPACS does not correlate with the Conju-Stru-VSA (vertical line from bottom to up).

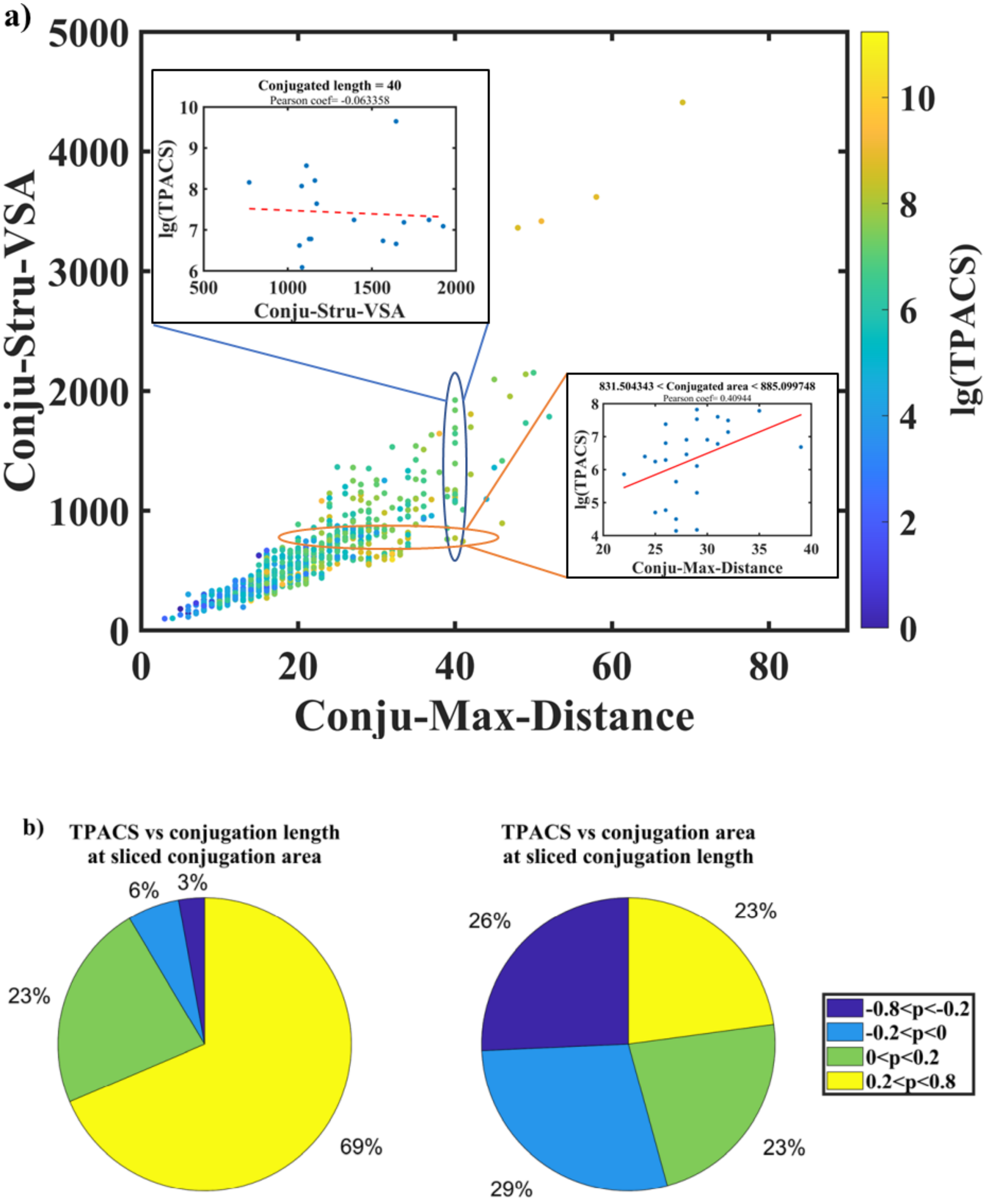
a)
Conju-Stru-VSA
Conju-Max-Distance
lg(TPACS)
Conjugated length = 40
Pearson coef= -0.063358
831.504343 < Conjugated area < 885.099748
Pearson coef= 0.40944
b)
TPACS vs conjugation length at sliced conjugation area
TPACS vs conjugation area at sliced conjugation length
69%
23%
6%
3%
26%
29%
23%
23%
-0.8<p<-0.2
-0.2<p<0
0<p<0.2
0.2<p<0.8

Figure S7. (a) Scatter plot of the distribution of conjugated length (Conju-Max-Distance) and conjugated area (Conju-Stru-VSA); markers are color-coded according to lg(TPACS). Inset figures showed Pearson coefficients of a horizontal and a vertical slice of the samples, respectively. (b) Pie plots of the Pearson coefficient distribution. Pearson coefficient was represented by p in the legend.

To be more quantitative in isolating the contribution of these two features, the data were sliced into 35 pieces twice, first vertically (slice on the Conju-Max-Distance axis) and then horizontally (slice on the Conju-Stru-VSA axis) to control the value of Conju-Max-Distance or Conju-Stru-VSA in a subset. We examined the relationship between the Conju-Stru-VSA and the TPACS in the subset slice of similar Conju-Max-Distance values using Pearson coefficient. The distributions of the Pearson coefficients over the 35 subsets are shown in Figure S7b(right). On the other hand, we performed similar analysis between the Conju-Max-Distance and the TPACS in the slice controlling Conju-Stru-VSA and show the Pearson coefficient distribution in Figure S7b(left). The result clearly showed a strong correlation between the Conju-Max-Distance and the TPACS but a poor correlation between the Conju-Stru-VSA and the TPACS.

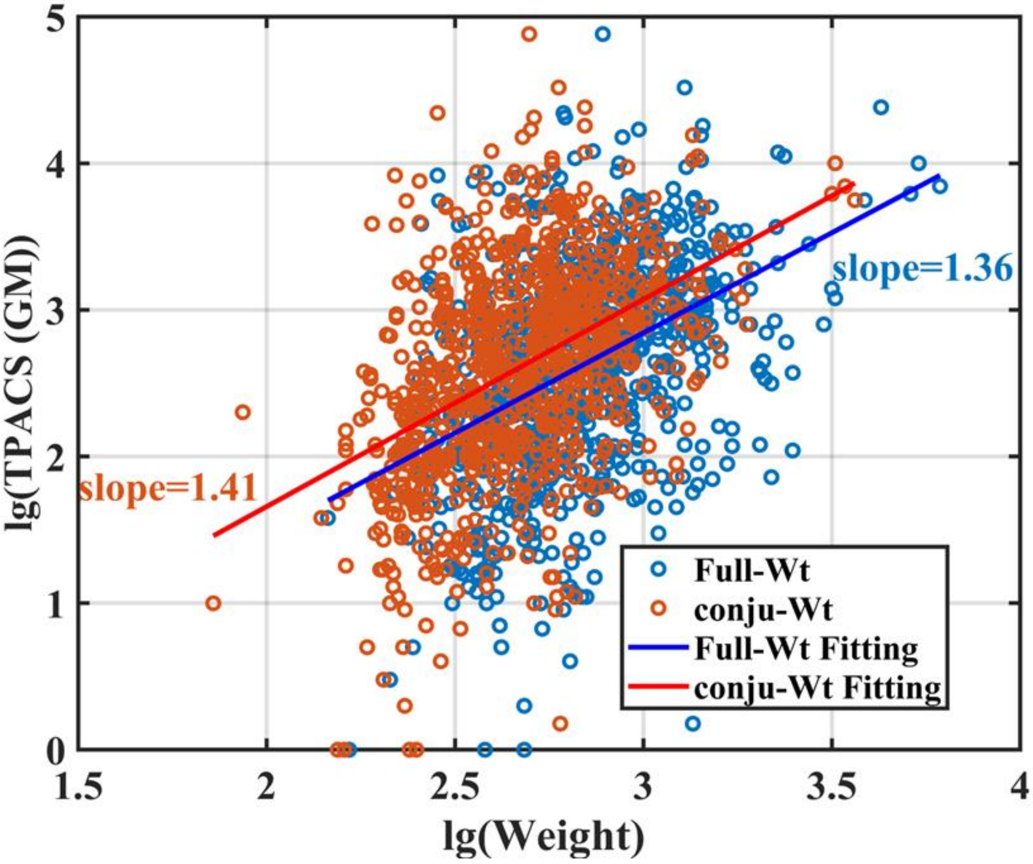

Figure S8. Scatter plot and correlation between TPACS and molecular weight in the log scale

Group	Molecules	Conj-Max-Distance	TPACS/GM
3	579	17	330
3	580	25	200
3	581	33	330
3	582	40	780
3	583	28	170

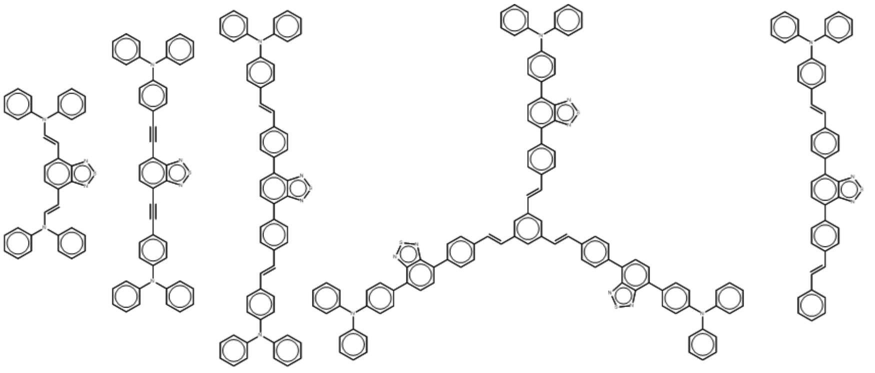

579 580 581 582 583

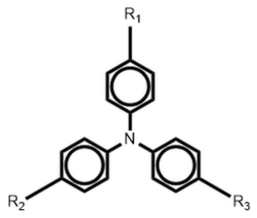

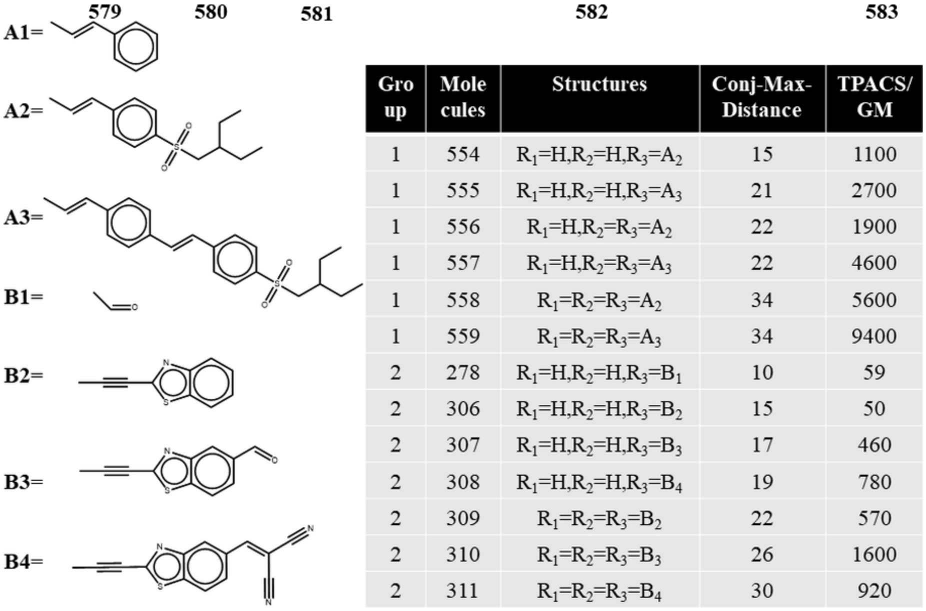

Group	Molecules	Structures	Conj-Max-Distance	TPACS/GM
1	554	R_1=H,R_2=H,R_3=A_2	15	1100
1	555	R_1=H,R_2=H,R_3=A_3	21	2700
1	556	R_1=H,R_2=R_3=A_2	22	1900
1	557	R_1=H,R_2=R_3=A_3	22	4600
1	558	R_1=R_2=R_3=A_2	34	5600
1	559	R_1=R_2=R_3=A_3	34	9400
2	278	R_1=H,R_2=H,R_3=B_1	10	59
2	306	R_1=H,R_2=H,R_3=B_2	15	50
2	307	R_1=H,R_2=H,R_3=B_3	17	460
2	308	R_1=H,R_2=H,R_3=B_4	19	780
2	309	R_1=R_2=R_3=B_2	22	570
2	310	R_1=R_2=R_3=B_3	26	1600
2	311	R_1=R_2=R_3=B_4	30	920

Figure S9. Three groups of selected molecules

Section 7. Accumulated Local Effects

To further validate the quantitative dependence, another analysis method, accumulated local effects (ALE)was used. The ALE plot of a given feature was obtained by evaluating the change of the predicted lg(TPACS) upon changing the feature value. The ALE values are centered at zero and correspond to the deviation of lg(TPACS) of a given sample from the mean value due to the contribution of a particular feature. The ALE is linear to lg('Conju-Max-Distance') with a slope of 2.2±0.1 (Figure S10a).

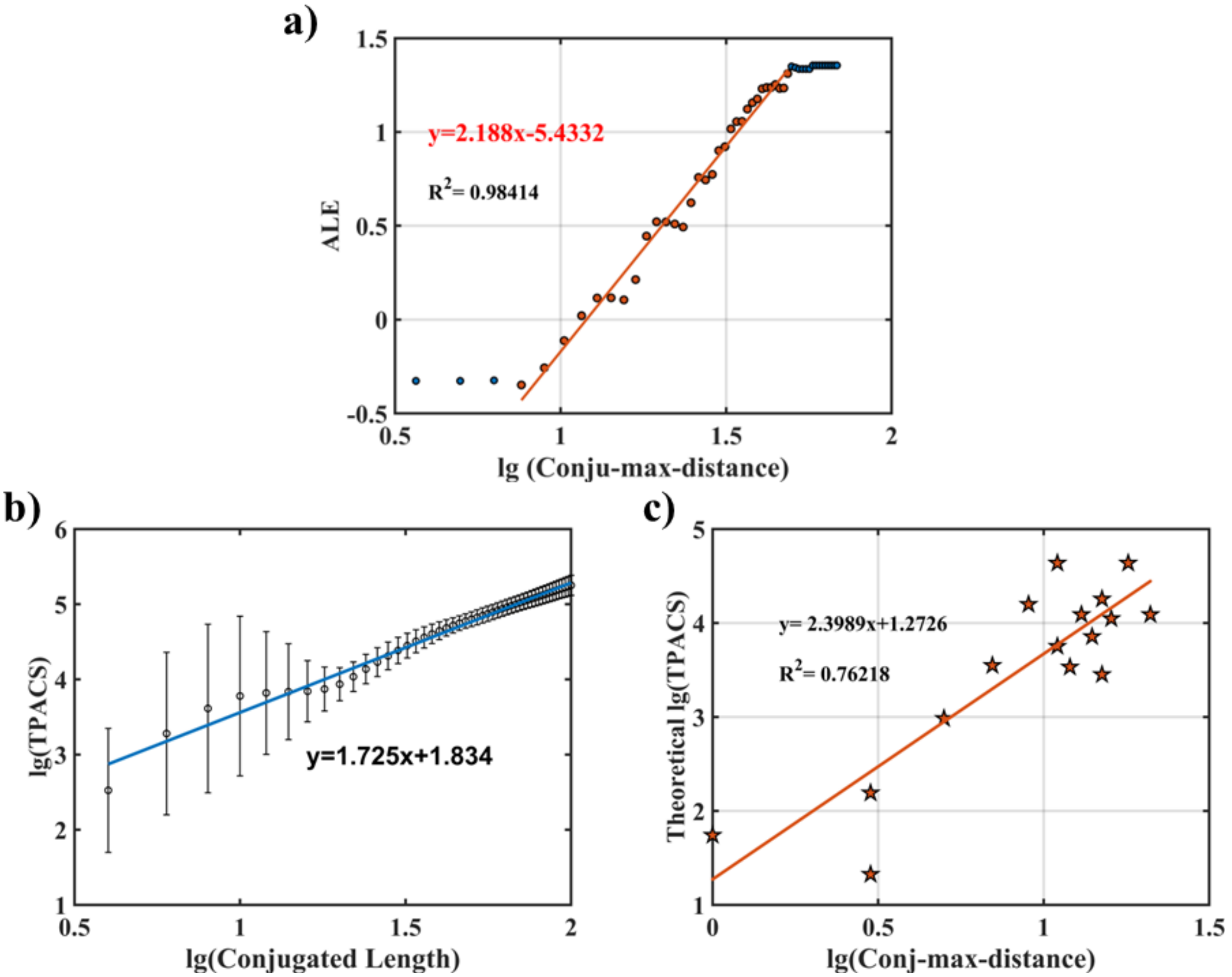

Figure S10. (a) ALE plot of Conju-Max-Distance. (b) Conjugation length dependence of TPACS simulation using a model of alternative double and single bonds. (c) Simulation results of several simplified molecular models.

Section 8. Model 1D conjugation system

A simple model of conjugated parallel *p*-orbitals with alternative double and single bonds is shown below. A Hamiltonian was obtained considering couplings between only adjacent *p* orbitals. The coupling constants between the two *p* orbitals in the alternative single bond and double bond are set to be different with a ratio of 0.8. These two coupling constants are thus set to be 0.8J and J (J is a tuning parameter). Electron donor and acceptor can be introduced at the two ends of the linear model by changing the diagonal energy element of the Hamiltonian at the

first and/or the last position. So the Hamiltonian considering couplings between only adjacent p orbitals could be written as:

$$\hat{H} = \begin{pmatrix} E_D & J & 0 & & 0 & 0 & 0 \\ J & 0 & 0.8J \ldots & & 0 & 0 & 0 \\ 0 & 0.8J & 0 & & J & 0 & 0 \\ & \ldots & & & & \ldots & \\ 0 & 0 & J & & 0 & 0.8J & 0 \\ 0 & 0 & 0 & \ldots & 0.8J & 0 & J \\ 0 & 0 & 0 & & 0 & J & E_A \end{pmatrix},$$

where E_D is the orbital energy of the donor; E_A is the orbital energy of the acceptor; the number of electrons is $2n$.

Diagonalizing this Hamiltonian can give a series of orbitals.

By assuming every p orbital contributing one electron, we selected the HOMO−1, HOMO, LUMO, LUMO+1 orbitals to be the $n-1$, n, $n+1$, $n+2$ orbitals, respectively. We used these four orbitals to construct five Slater determinants to represent a ground state and four excited states, ignoring configuration interactions.

We calculated the transition dipole moments between these states in an arbitrary unit and calculated TPACS according to

$$\sigma_{TPA} \propto \sum_m \frac{\mu_{gm}^2 \mu_{mf}^2}{\left[E_{mg} - \frac{E_{gf}}{2}\right]^2} + \frac{\mu_{gf}^2 (\mu_{ff}^2 - \mu_{gg}^2)}{\left[\frac{E_{gf}}{2}\right]^2},$$

where σ_{TPA} is the TPA cross section, μ_{ij} is the transition dipole moment between the two states i, j or the dipole moments of the ground or excited state when i=j; E_{ij} is the energy difference between these two states. g stands for the ground state; f stands for the final state; m stands for a mediating state in the TPA process based on the second order perturbation theory.

The TPA cross section of such a model was then calculated using numerical simulations. We are interested in the slope of lg(TPACS) *vs* lg(n).

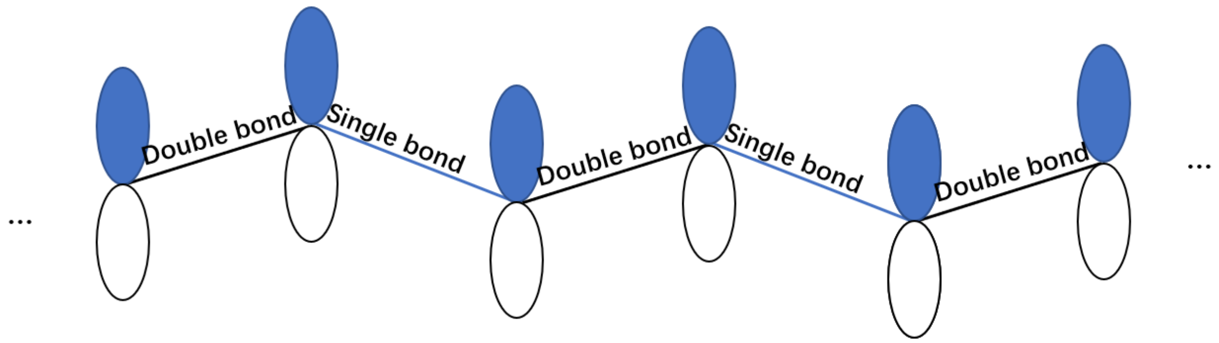

Figure S11. Model 1D conjugation system with alternative bond lengths.

Section 9. TDDFT Computational Details

The Gaussian 16 program package was used to calculate the transition dipole moment between states by time-dependent density functional theory (TDDFT) using the CAM-B3LYP functional, aug-cc-pVDZ basis set after all geometries were optimized at wB97xD/def2-TZVP level. All of two-photon absorption spectrum simulations were performed by a code using the MATLAB program used in our previous work[7]. The transition dipole moment matrix and the oscillator strengths had been prepared by TDDFT calculations and extracted by Multiwfn. We calculated the TPACS using sum-over-states (SOS) method using the lowest 20 singlet excited states.[7-8]

Table S5. Molecular structures and their simulated TPACS using TDDFT.

Structures	Conj-max-distance	Theoretical lg(TPACS)	Structures	Conj-max-distance	Theoretical lg(TPACS)
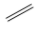	1	1.74	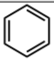	3	0.48
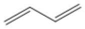	3	2.19	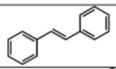	15	1.18
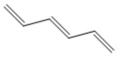	5	2.99	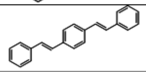	21	1.32
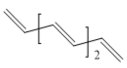	7	3.55	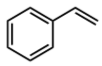	5	0.70
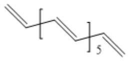	9	4.20	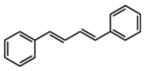	11	1.04
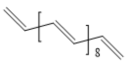	11	4.64	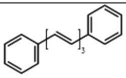	13	1.12
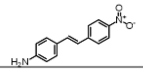	12	3.53	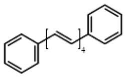	15	1.18
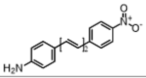	14	3.86	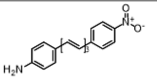	16	4.05

Section 10. Principle component analysis

The "MR-Max" is correlated to the conjugation length. To avoid misinterpretation of this feature, we used principal component analysis (PCA) to reorganize the six critical features and rerun the SHAP analysis. The six PCA components each correspond to one of the six features as only one feature has the highest contribution to each component with a certain degree of mixing. The mixing among the features are shown in Table S6.

Table S6. Principal component analysis. The rows contain the coefficients for the 6 features, and its columns correspond to 6 principal components.

Raw Feature Names	New feature 1	New feature 2	New feature 3	New feature 4	New feature 5	New feature 6
Max-Conju-Distance	0.000	0.445	-0.499	0.744	-0.002	0.002
PEOE-Charge-Max	0.001	-0.004	0.003	0.000	-0.404	0.915
LogP-Min	-0.001	0.006	-0.005	-0.005	0.915	0.404
MR-Max	-0.014	0.888	0.353	-0.294	-0.006	0.000
ET(30) (Solvent)	0.009	-0.116	0.791	0.601	0.008	0.000
Wavelength (Exp nm)	0.999	0.014	-0.002	-0.009	0.001	0.000

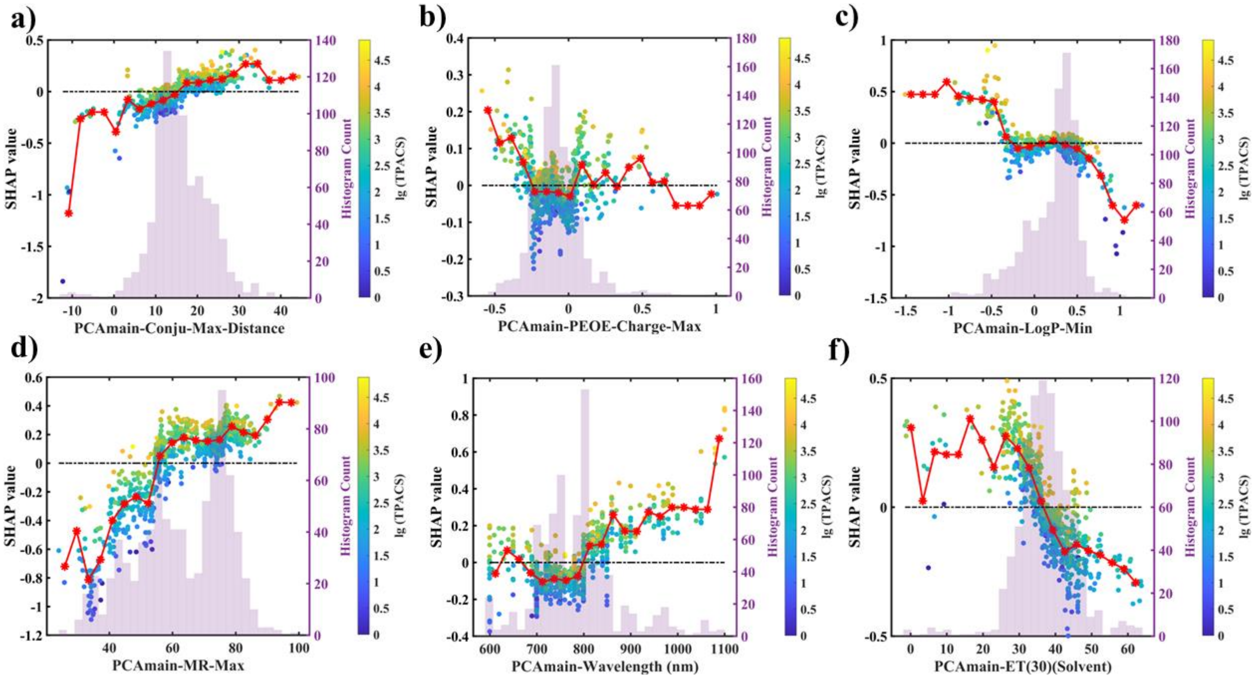

Figure S12. SHAP Feature Contribution of MFF-MOE features after principal component analysis (PCA). The main component of new features was shown in X label

Section 11. Interpreting "MR-Max"

The mixing of conjugation length to the "MR-Max" is shown in the column "New Feature 2" in Table S6. Consistent with the notion of the correlativity, the SHAP plot of "PCAmain-MR-Max" after PCA (Figure S12d) changed significantly from the original one, while the SHAP of other features did not change much. The SHAP plot showed that the TPACS is positively correlated to the "PCAmain-MR-Max".

We drew several MFF structures with extreme feature values to probe the chemical information of this feature (Figures S12c,d). All the structures are the conjugation backbones. The conjugated structure connected through carbon-carbon double bond, triphenylamine groups seem to increase the SHAP value, while a direct connection between two aromatic rings via a single bond has a negative effect. This later observation can be easily verified by analyzing the number of 'ccc(cc)-c(c)c' MFF in molecule as a feature (Figure S14c), which showed negative SHAP values to the TPACS. These connections affect coplanarity of the conjugation system. In the single bond linkage between two benzene rings, the steric repulsion of adjacent hydrogen atoms leads to a dihedral angle of ~60° that decreases the degree of conjugation.

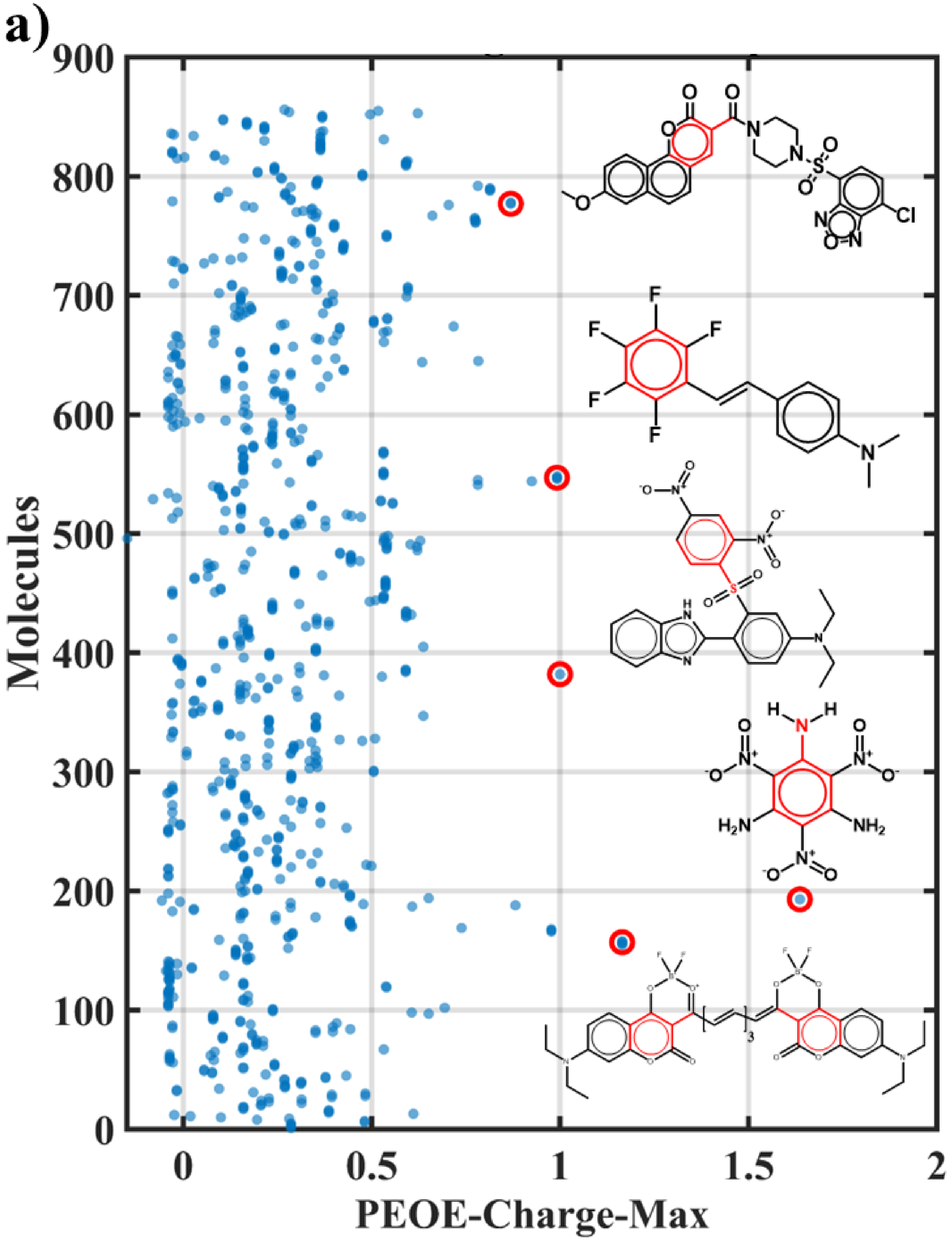
a)
900
800
700
600
500
400
300
200
100
0
Molecules
0
0.5
1
1.5
2
PEOE-Charge-Max

b)

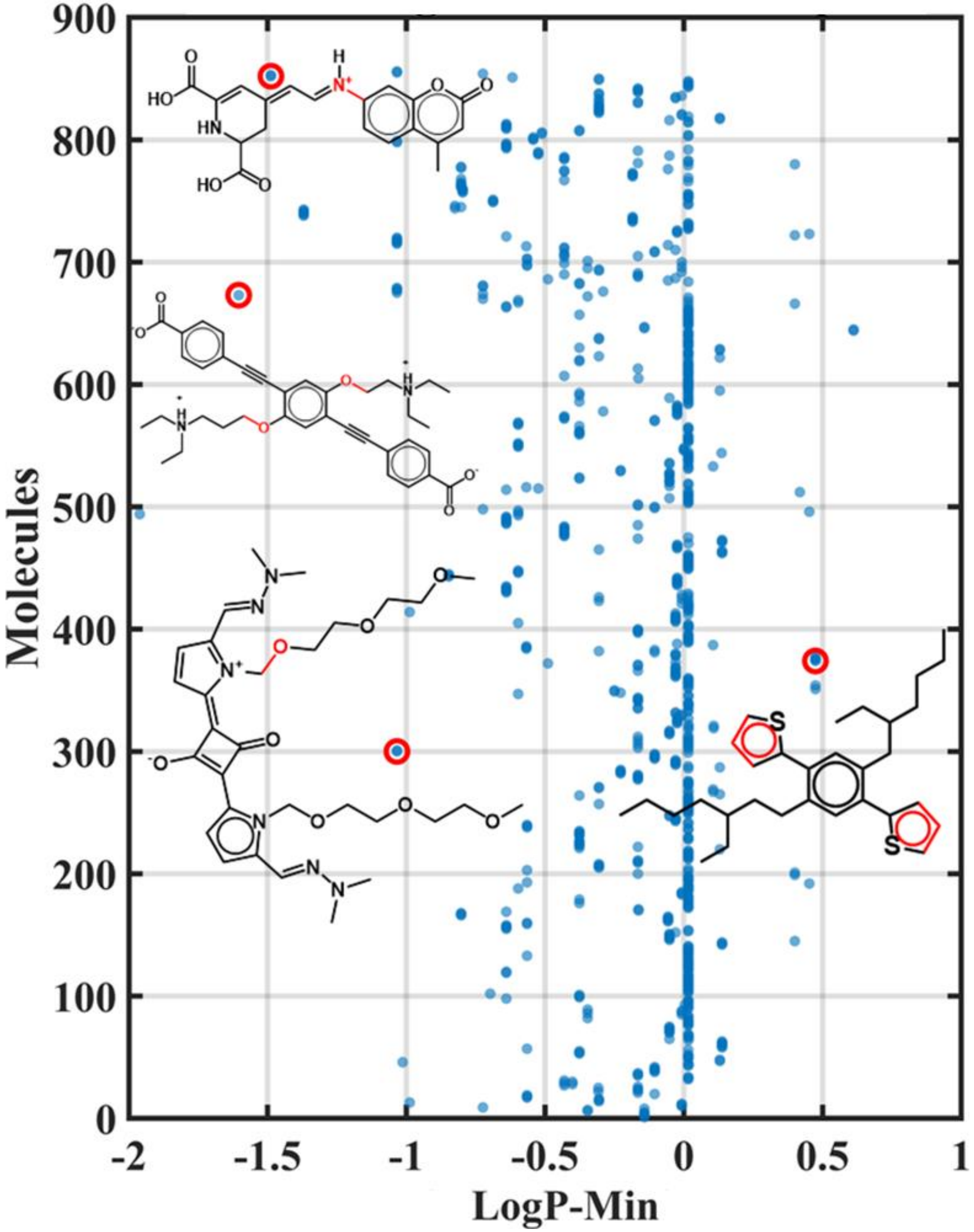
900
800
700
600
500
400
300
200
100
0
Molecules
-2
-1.5
-1
-0.5
0
0.5
1
LogP-Min

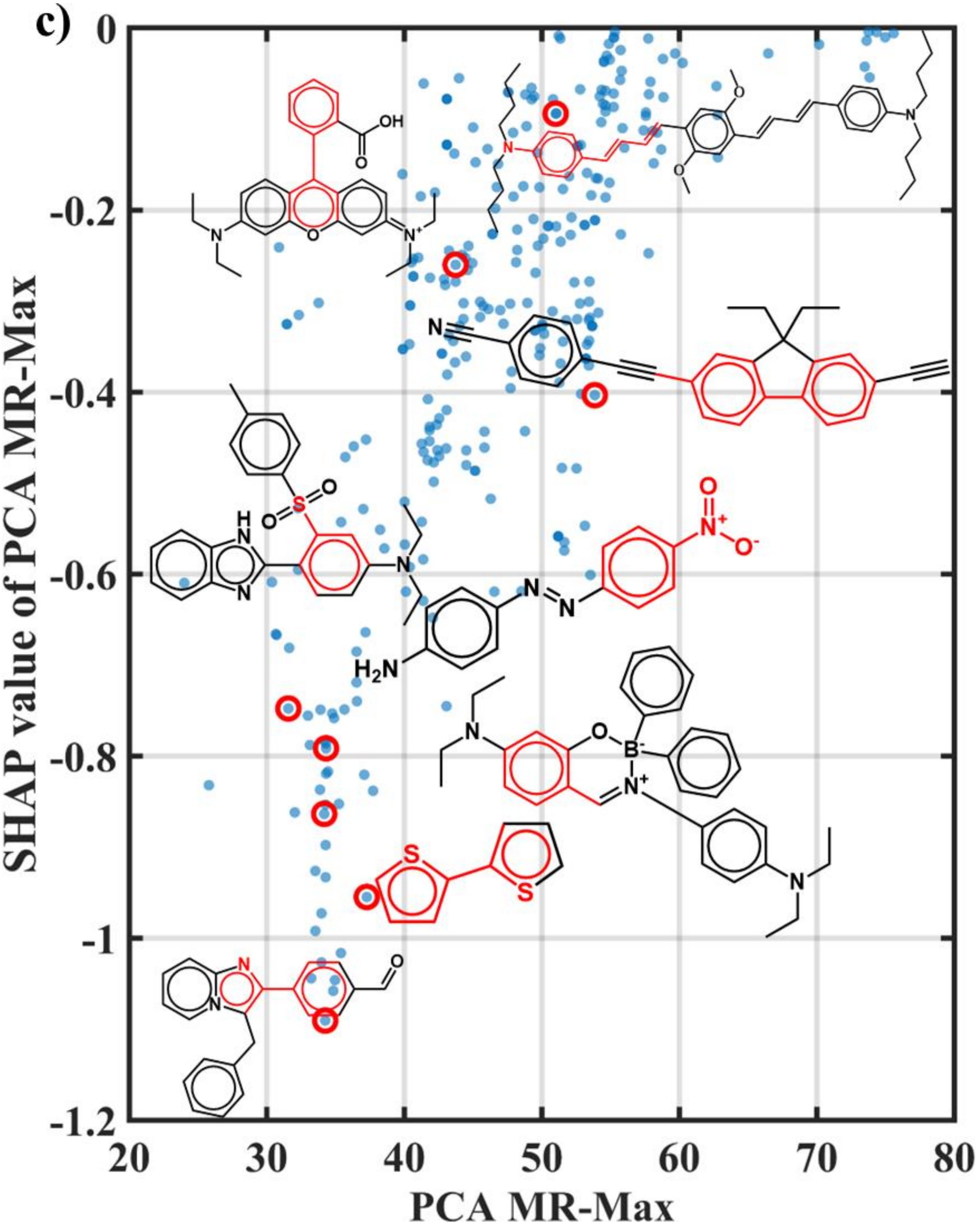
c)
0
-0.2
-0.4
-0.6
-0.8
-1
-1.2
20
30
40
50
60
70
80
PCA MR-Max
SHAP value of PCA MR-Max

d)

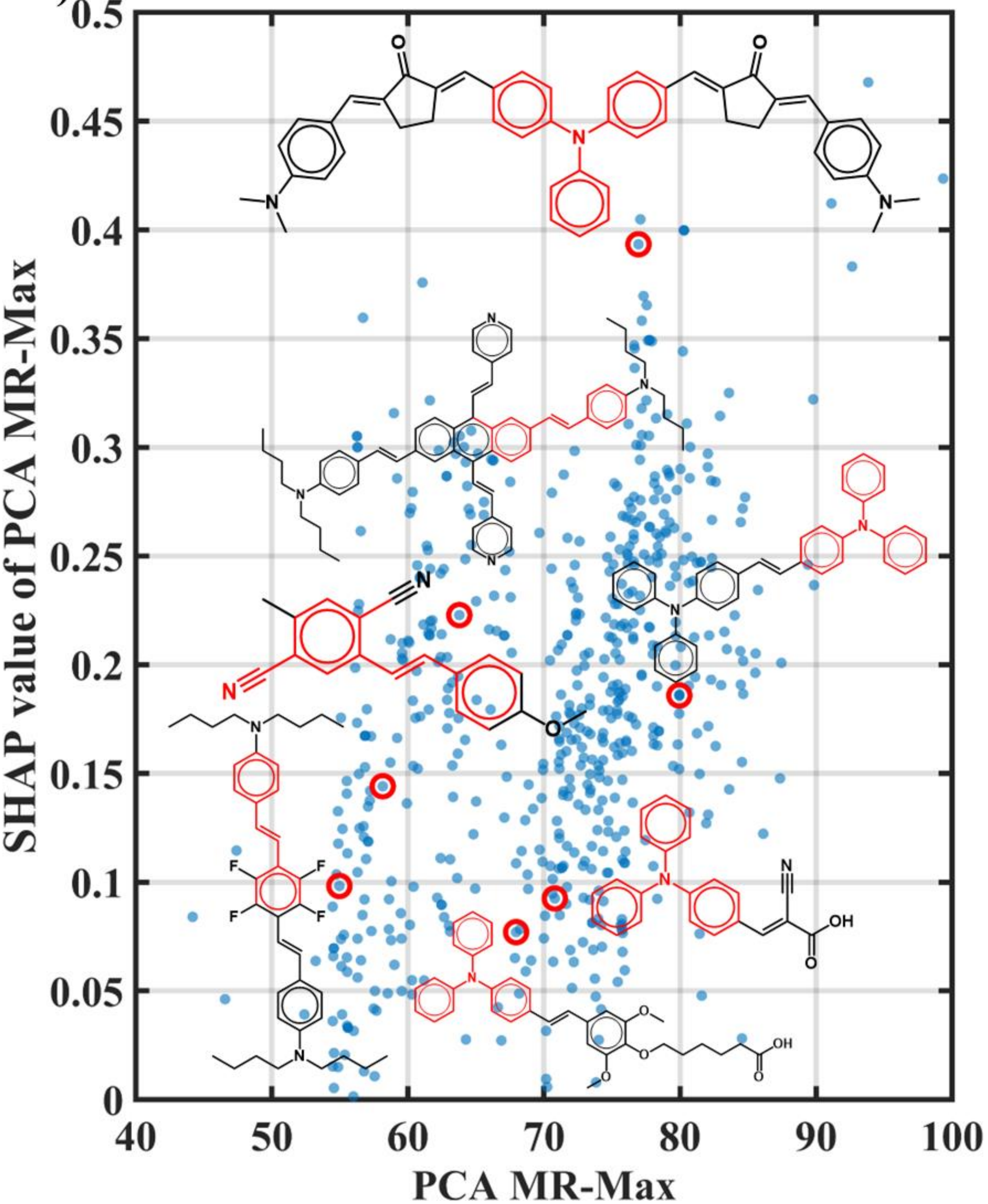

SHAP value of PCA MR-Max
PCA MR-Max
0
0.05
0.1
0.15
0.2
0.25
0.3
0.35
0.4
0.45
0.5
40
50
60
70
80
90
100

Figure S13. (a)–(b) Scatter plot of the distribution of the MFF-MOE features and illustrative examples. (c)–(d) SHAP values of the MR-Max feature after PCA and illustrative examples. Red circle represents the selected molecules as examples. Red fragments highlighted in the molecules represented the fragments selected by the MFF-MOE algorithm.

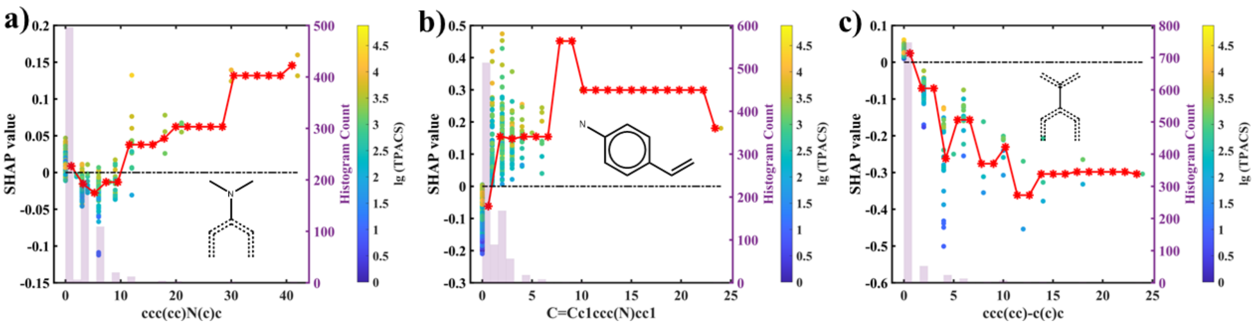

Figure S14. SHAP Feature Contribution of some MFF descriptors of interest.

Section 12. Features describing multipolar and quadrupolar structures

Conju-Branch-Ratio

To isolate the effect of branching, we proposed a conjugated descriptor called 'Conju-Branch-Ratio' (Figure S15a) that is only weakly correlated to the conjugation length and able to tell the difference between linear and branched structures. The 'Conju-Branch-Ratio' is close to 1 when the branching point appeared in the middle of a conjugated system, while it is close to 0 for a linear structure.

The Conju-Branch-Ratio is more suitable to our task of analyzing multipolar effect than several other structural shape features from the RDKit library ("Kappa3", "Chi3n", and "Conju-Stru-Wiener-Index"), as the latter three are all strongly correlated to the conjugation length (Figure S15).

DAratio

The DAratio measures the distance between the donor and acceptor groups in a conjugated system by finding the distance between centers with the most positive and most negative PEOE charges. This distance is then divided by the conjugation length to give the "DAratio". A "DAratio" close to 0.5 corresponds to D-A-D or A-D-A structure, while a "DAratio" close to 1 corresponds to a D-A structure. A "DAratio" close to 0 indicates either very close D-A groups or the lack of any D or A groups.

Triphenylamine core

The triphenylamine core has a positive effect to increase TPACS as shown by the SHAP plot in Figures S14a-b, which happens to be a branching core and partly contributes to the impression of beneficial branching effect.

This triphenylamine core has many reasons to be a designer structure motif for obtaining high TPACS: (1) it is a strong electron-donating group itself; (2) it is part of the conjugation backbone to ensure a large conjugation length; (3) it bridges three benzene rings to ensure a high degree of conjugation although the benzene rings are not coplanar. These different aspects of the triphenylamine core are already captured by the selected four molecular features.

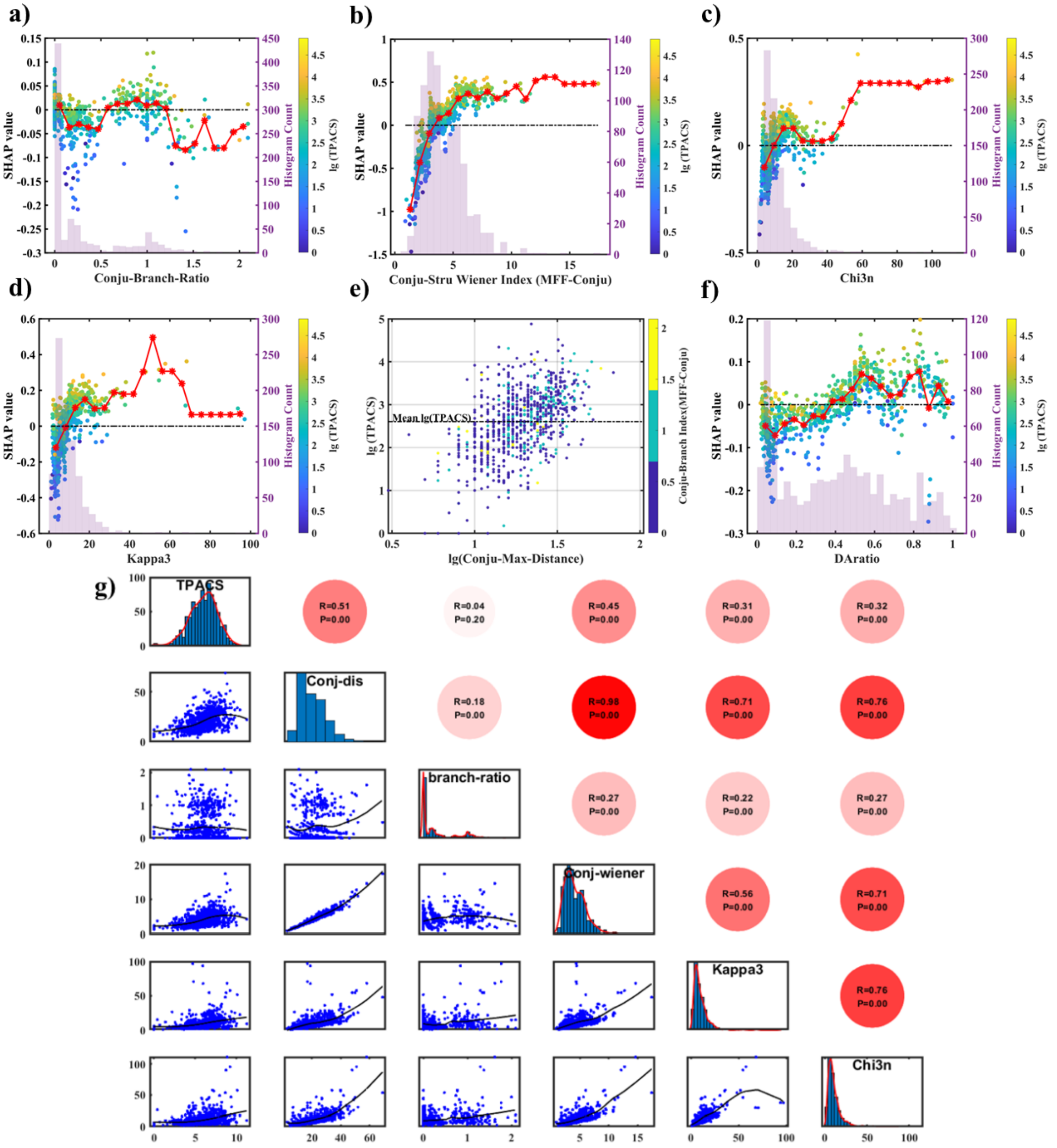

Figure S15. (a-f) SHAP feature contribution of branching-related features. (g) Correlation scatter matrix of branching-related features and the TPACS with correlation coefficients in upper triangle and scatter plots in the lower triangle. The diagonal line is histogram with nonparametric kernel-smoothing distribution. The R value is Pearson's linear correlation coefficient, and the p

value is used for testing the hypothesis of no correlation against the alternative hypothesis of a nonzero correlation. The smaller p value, the larger and darker the red circle.

a)

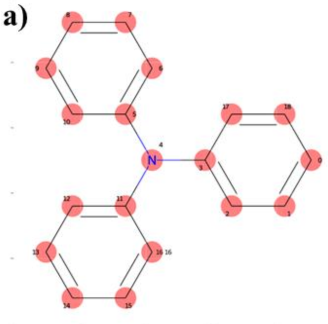

L_{conj} =[0 0 8]
Conju Max Distance: 8.0
Conju-Branching Index:1
DAratio:0.125

b)

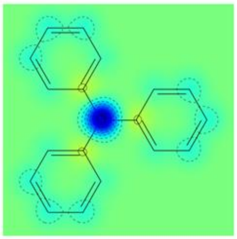

c)

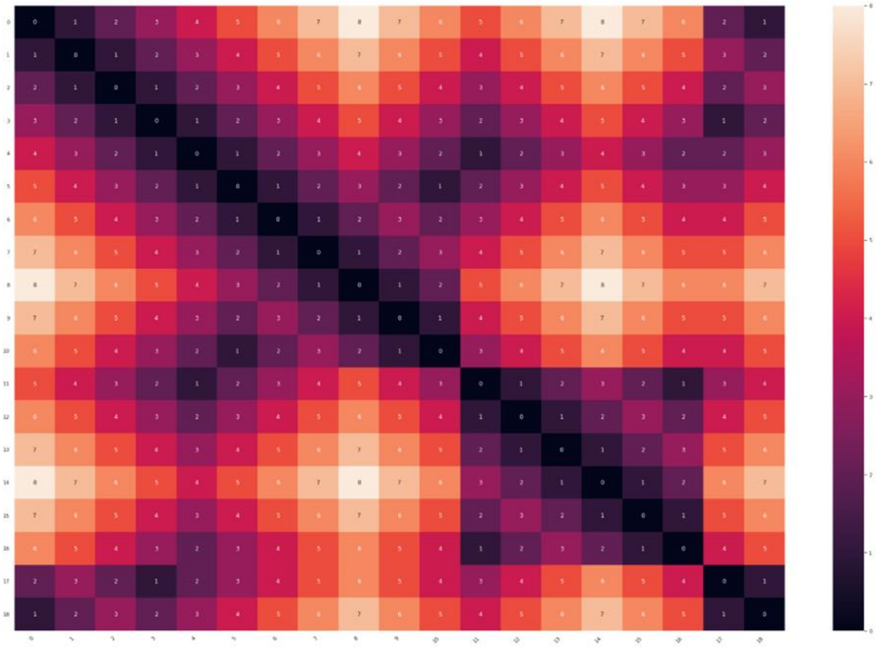

a)

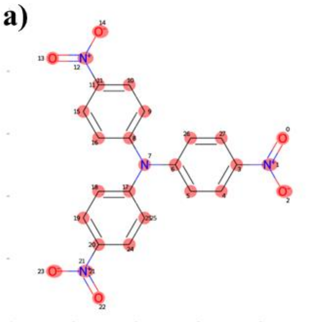

L_{conj}=[0 0 0 0 2 2 2 2 13 13 13 13]
Conju Max Distance: 12.0
Conju-Branching Index: 1.08333333
DAratio:0.3333333333333333

b)

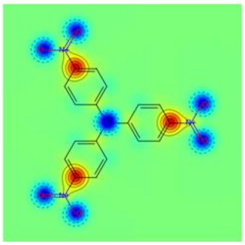

c)

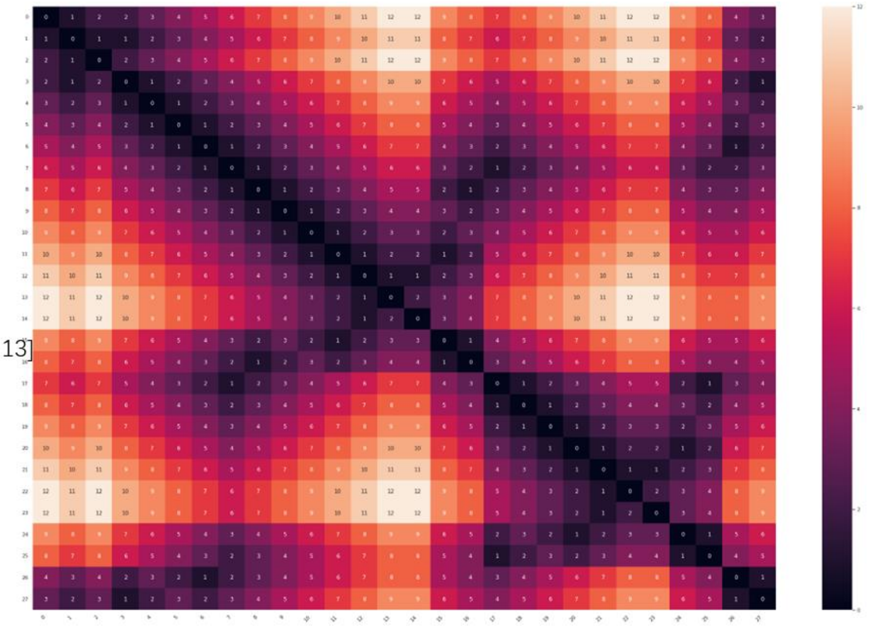

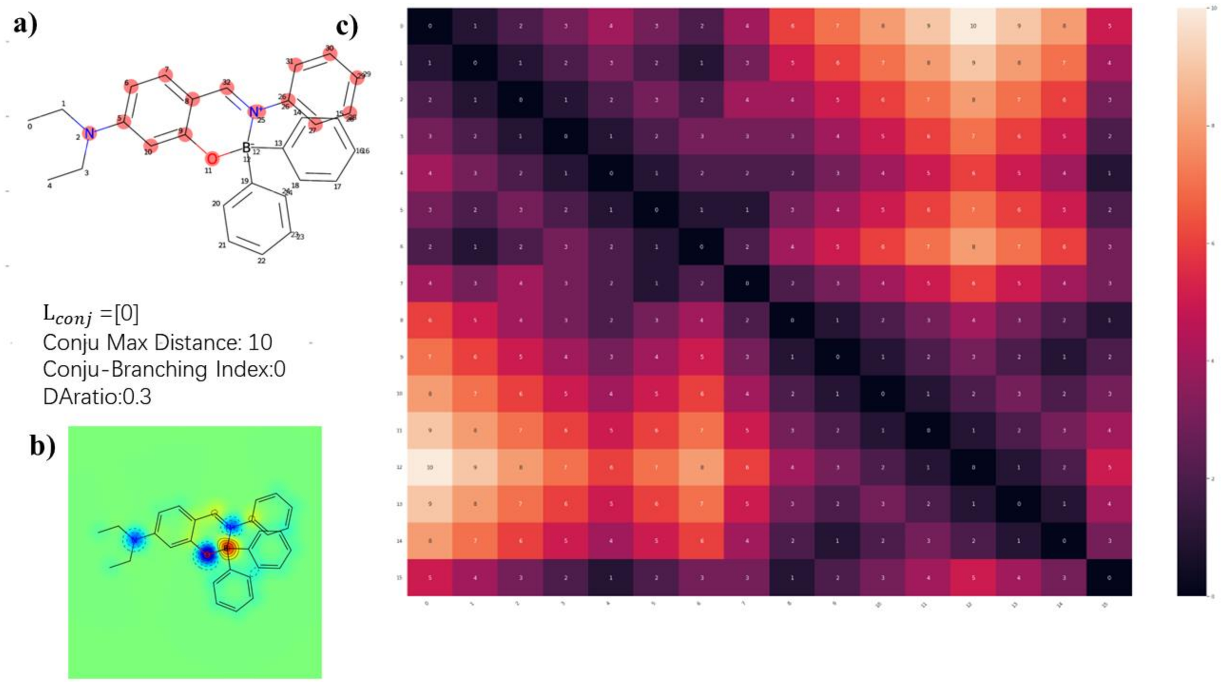

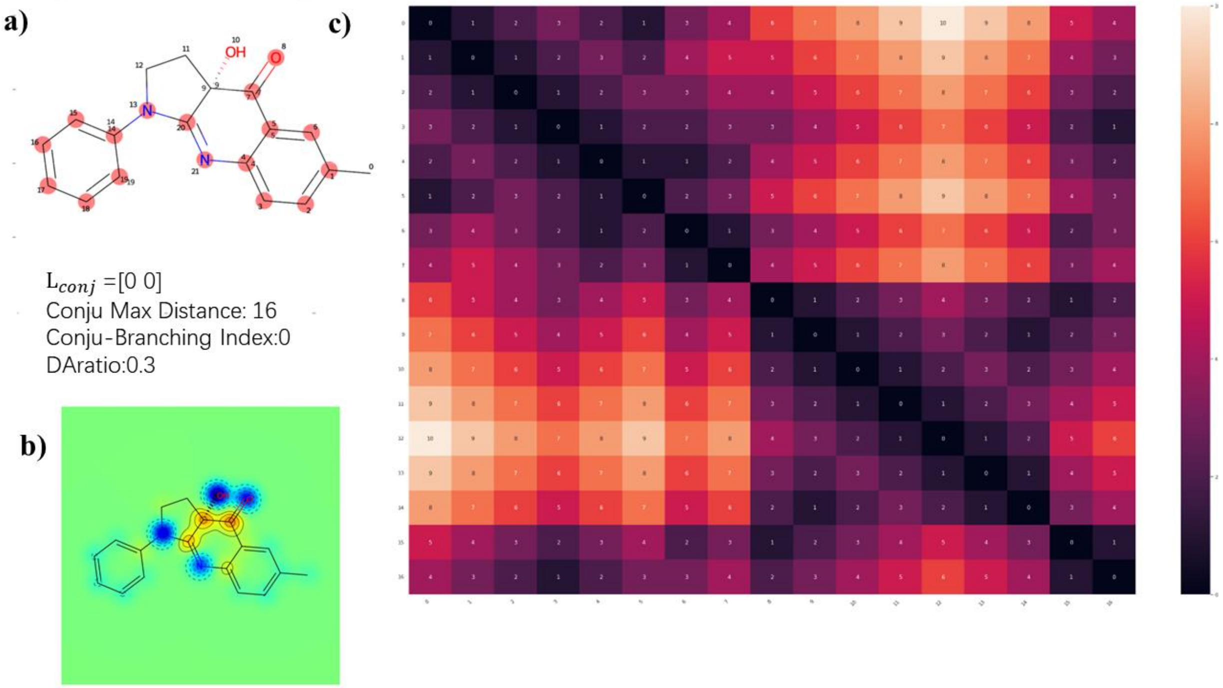

Figure S16. Four examples illustrating the Conju-Max-Distance, Conju-Branch-Ratio, DAratio. (a) Chemical structure and atom index of selected molecules. Conjugated structures are highlighted with red cycle. (b) The PEOE charge of atoms are colormapped. Blue refers to negative values, while red refers to positive values. (c) The distance matrix of conjugated structures of the selected molecule. The number and color in every block represent the distance between two atoms.

Section 13. Analysis of other features

Is aliphatic chain helpful?

A structural component descriptor from RDKit, 'FractionCSP3' (Figure S17) describes the fraction of sp^3 hybrid C atoms in all C atoms of one molecule. An obvious negative SHAP value of this descriptor is observed after the fraction of the sp^3 carbon is beyond 0.5, which can be understood as too many sp^3 hybrid C atoms indicate small conjugated parts in the molecule. The optimum FractionCSP3 value is not at zero but at around 0.2-0.4, which suggests that some aliphatic chains on the molecule are beneficial. This may work by improving the solubility and solvation of the molecules. However, adding this feature to the feature matrix does not improve the overall performance of the model, possibly due to its small contribution.

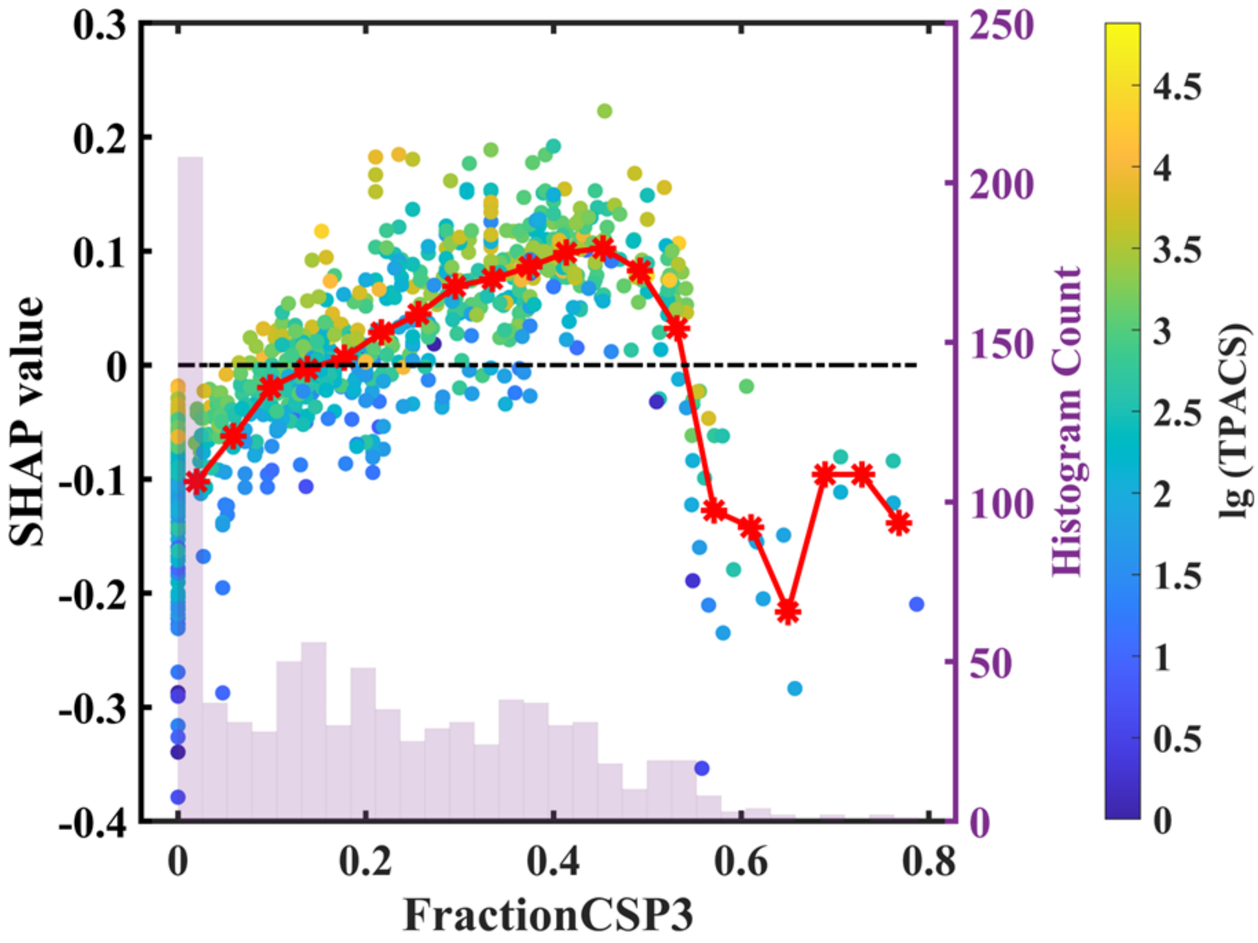

Figure S17. SHAP Feature Contribution of Descriptors based on RDKit.

Is there any significant effect from the solvent or test method used in experiment?

The descriptor of the polarity of the solvent, 'ET(30)' (Figure 4f) shows that the solvent used in TPA testing would only make a small contribution to the testing results. A larger ET(30) value representing a more polar solvent gives a slightly more negative contribution to the lg(TPACS). The dependence on the testing wavelength (Figure 4e) showed that longer wavelength correlated with higher TPACS.

We also counted the measurement methods used, the majority of which are Z-scan and two-photon excited fluorescence (TPEF) in Figure S18. Similar TPACS distributions were observed

for the two methods but the sets of molecules with the highest TPACS were mostly measured by Z-scan, possibly because their emission quantum efficiencies are too low to detect their fluorescence, due to the energy gap law.

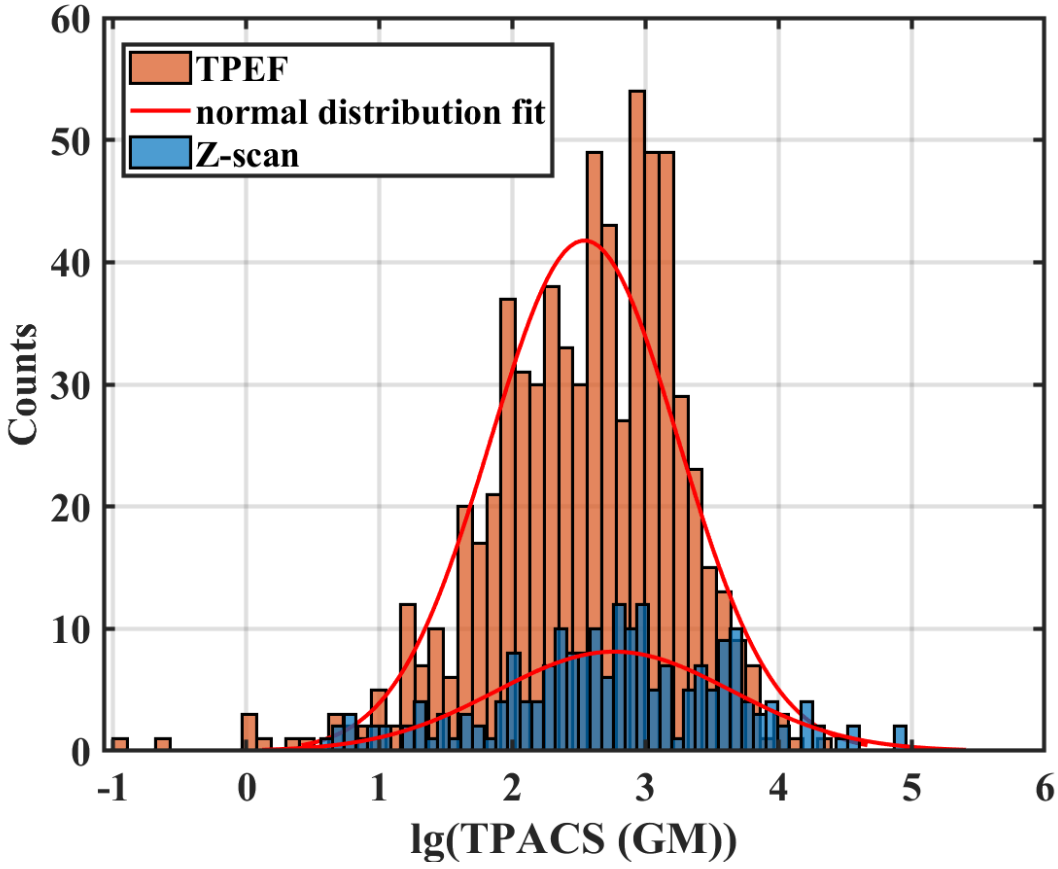

Figure S18. Distribution of TPACS measured by the Z-scan and TPEF methods.

We carried out a statistical analysis called Kolmogorov–Smirnov test (*Statistical Math.* **2001**, 54, 3), to check whether the distributions using the Z-scan and TPEF are alike or different. This test gives a p-value of 3.47×10^{-5}, which is significantly smaller than 0.05, suggesting that the distributions of TPACS measured by Z-scan and TPEF are significantly different.

This difference between the two methods is expected, as molecules without fluorescence cannot be measured by the TPEF method. As a result, molecules with very large conjugation

system that tend to have high TPA cross sections also have small energy gaps and thus low fluorescence quantum yields. The TPEF methods are thus intrinsically biased towards low TPA cross section.

Section 14. Experimental Validation

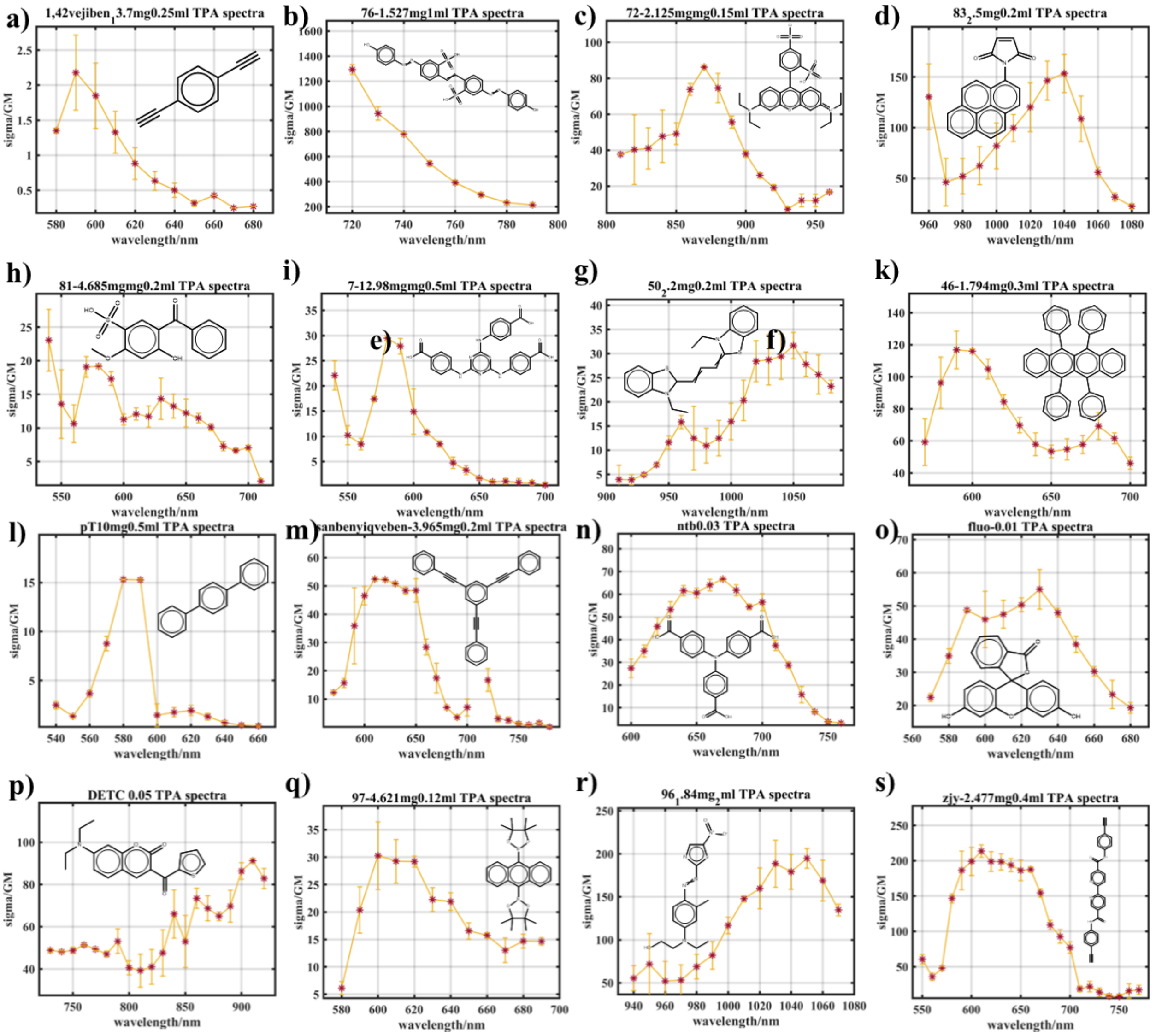

Figure S19. 16 newly measured molecules by Z-scan method.

Z-scan measurements were performed with a home-built equipment based on a commercial titanium: sapphire amplifier (Coherent Legend Elite HE+ USP-1k-II, 800nm, 5mJ/pulse, 1 kHz,

35 fs). During the Z-scan experiment, the nonlinear extinction coefficient (ε^{NL}) could be calculated by curve fitting to the open aperture traces with the following equation:

$$T(z) = \sum_{m=0}^{\infty} \frac{\left[{}^{-\varepsilon^{NL} I_0(t) L_{eff}}\Big/_{(1 + Z^2/Z_0^2)}\right]^m}{(m+1)^{1.5}}$$

where L_{eff} = (1 − e−$\alpha L/\alpha$), I_0 is the peak intensity, Z_0 = $\pi w_0{}^2/\lambda$, w_0 is the radius of light beam, Z is the relative position with the focal plane, m is the number of recursive items. We summed over m=0 to 20 for practical calculations. After fitting to the experimental data, the value of ε^{NL} is obtained to derive the value of TPACS. TPACS could be calculated from the nonlinear extinction coefficient ε^{NL}.

$$h v \varepsilon \mathrm{NL} = \mathrm{TPACS} \times N_A \times d \times 10^{-3}$$

where h is the Planck constant, v is the light frequency, N_A is the Avogadro constant, d is the concentration of the solution (mol/L).

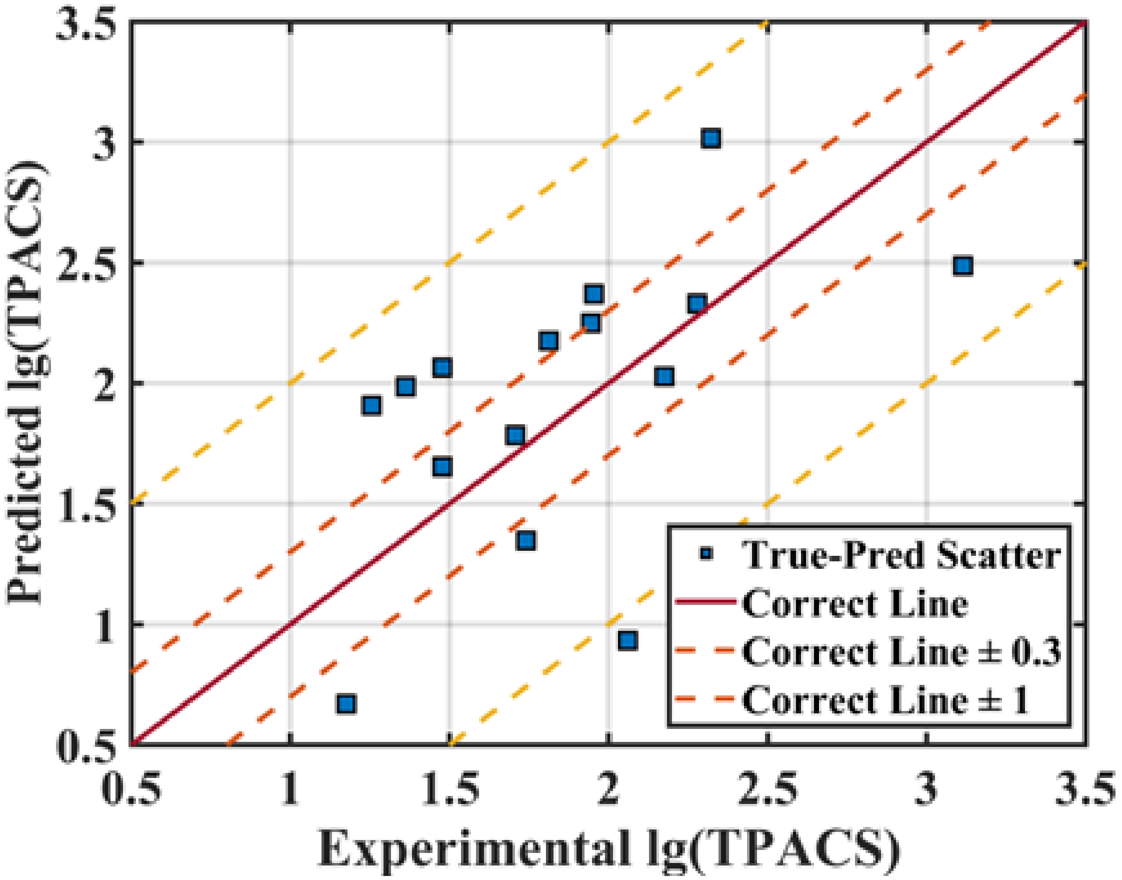

Figure S20. Scatter plot for experimental and ML predictions of lg(TPACS) of 16 test molecules with their two-photon absorption spectra measured in this work.

Section 15. Comparison between the machine learning performance with and without QM descriptors

Molecules in this dataset were geometry-optimized and descriptors like excitation energy and HOMO-LUMO gap were calculated using time-dependent DFT approach. ZINDO is also adopted to calculate some of the same quantities. Furthermore, some interpretable descriptors based on electron-hole analysis and electrostatic potential analysis by using Multiwfn software were also put into the feature matrix. The structure optimization was first performed by xtb and then refined by Gaussian using B3LYP(D3)/6-31g*. TDDFT calculation was implemented using PBE0/6-31g*.

Exactly the same pipeline of machine learning procedure was employed to extract important features. 120 splits of training and testing sets were randomly generated to evaluate the model performances with a train-test ratio of 85:15. We combined the feature importance indexes of the three regressors (LASSO, GBRT and XGBOOST) into a weighted one (SI Section 3, Figures S1), which was used to remove the least important features one at a time from the feature matrix.

As a result, the performance of feature selection was shown in Figure S21, which showed that 20 features are sufficient to describe the system (Table S7) with the XGBoost performance of MAE of 0.38 and R^2 score of 0.48. Comparing with the performance based on MFF-MOE results, the QM-based machine learning is slightly less performing. (Table S8) The important features are listed below.

We prefer the use of the model without these QM descriptors as the QM-based models require additional calculations. Nevertheless, the QM-based results showed that the energy gaps and transition dipole moments are indeed significant, agreeing with the few-state models.

Table S7. Definition and top 20 ranking details based on QM descriptors. Calculation method and units were in the brackets.

Feature	Lasso Rank	GBRT Rank	XGBoost Rank
Energy Gap of S0-St2max (TDDFT)	1	1	1
Energy Gap of S0-St1max (TDDFT)	3	2	2
HOMO-LUMO Gap(xtb eV)	5	3	3
Orbital Delocalization Index(HOMO Gaussian Opt)	8	4	5
Negative Surface Area (Angstrom^2 Multiwfn ESP)	10	5	7
HOMO Energy(xtb eV)	7	12	6
Wavelength (nm)	20	11	4
Quadrupole Moment Magnitude(Gaussian Opt)	13	9	8
H CT (Multiwfn EHA)[a]	16	6	10
Negative Average Value (kcal/mol Multiwfn ESP)	2	10	11

Transition Dipole Moment in module of S0-St1max (ZINDO)	4	7	13
Composition of s Type of Shells(LUMO Gaussian Opt)	19	8	12
PosdivNeg Variance (Multiwfn ESP)	6	13	14
Energy Gap of S0-S1 (TDDFT eV)	17	19	9
RMSD of Electron (Multiwfn EHA)[b]	11	14	16
Transition Dipole Moment in module of S0-St1max (TDDFT)	14	17	15
Energy Gap of S0-St1max (ZINDO)	12	18	17
Absorbed Energy (eV)	9	15	20
Molecular Polarity Index MPI (Multiwfn ESP)	18	16	19
HOMO-LUMO Gap(eV TDDFT)	15	20	18

[a]H CT represents for the hole and electron extension along the direction of charge transfer. [b]RMSD of Electron represented for square mean root deviation of electron.

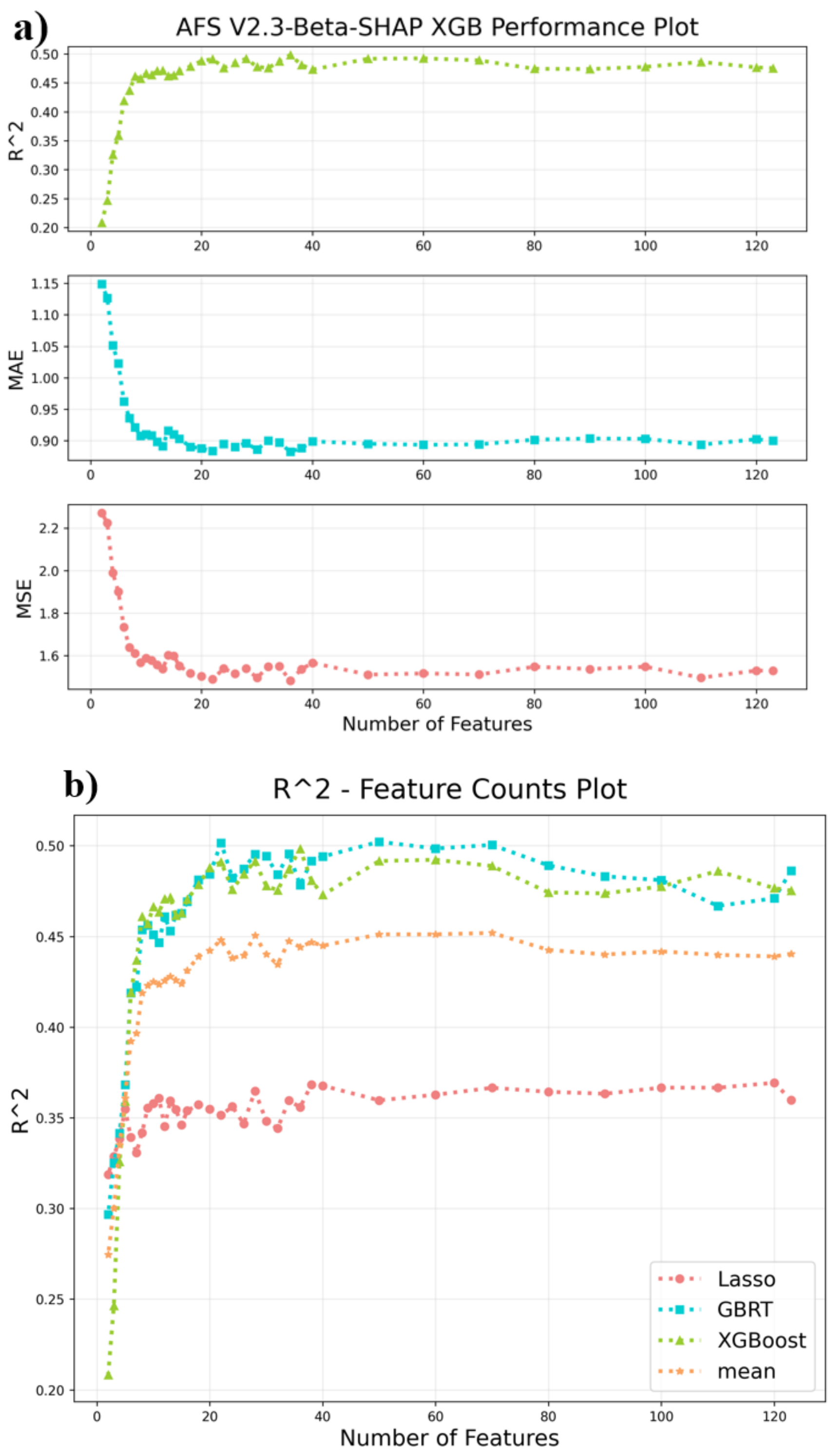

Figure S21. Model performance during feature selection procedure. (a) Mean squared error (MSE), mean absolute error (MAE) and R^2 score against feature selection procedure of XGBoost. (b) R^2 score of feature importance-based feature selection in LASSO, GBRT and XGBoost were denoted as red, blue and green, respectively.

Table S8. Performance of XGBoost regressor on QM based feature matrix.

Size	Origin of Features	Before feature selection		Reduced to top 20 features	
		MAE	R^2	MAE	R^2
856x696	MFF, RDKit, MFF-MOE	0.33	0.59	0.34	0.58
900x123	QM	0.39	0.47	0.38	0.48

Computational Details

xtb Program

Molecular geometries were initially optimized using GFN0-xTB implemented by xtb (6.4.1 version). The wave function analysis was done by Multiwfn, including orbital composition analysis, molecular structural analysis and dipole analysis.

ZINDO Program

ZINDO/S calculation was implemented by ORCA (4.21 version). Calculated by MNDO-AM1, there were 50 singlet excited states included. As a result, the information of the first state excitation energy, oscillator strength and transition dipole moment were calculated.

Geometry optimization and TDDFT calculation

Molecular geometries were fully optimized by Gaussian 09 at B3LYP(D3)/6-31g* level. TDDFT calculations were performed using the PBE0 functional and the basis set 6-31G* with Gaussian 09 version E.01. These calculations used Gaussian defaults with the exception of the keywords TD (nStates = 20 and IOp(9/40=4).

Electrostatic potential analysis[9]

we employed electrostatic potential (ESP) to intuitively describe electrostatic interaction characteristics. This analysis will provide us a general understanding on the basic character of intermolecular interaction and local polarity.

Hole-electron analysis [10]

In order to explore more useful descriptor of electronic excitations, we employed Multiwfn 3.8 to show a definitive picture about distribution of the hole and electron, which described where the excited electron leaves and arrives, respectively. To save computational resources, only the excited states of the brightest states of one-photon absorption (St1max) and two-photon absorption (St2max) were considered.

Section 16. The home-built Z-scan equipment

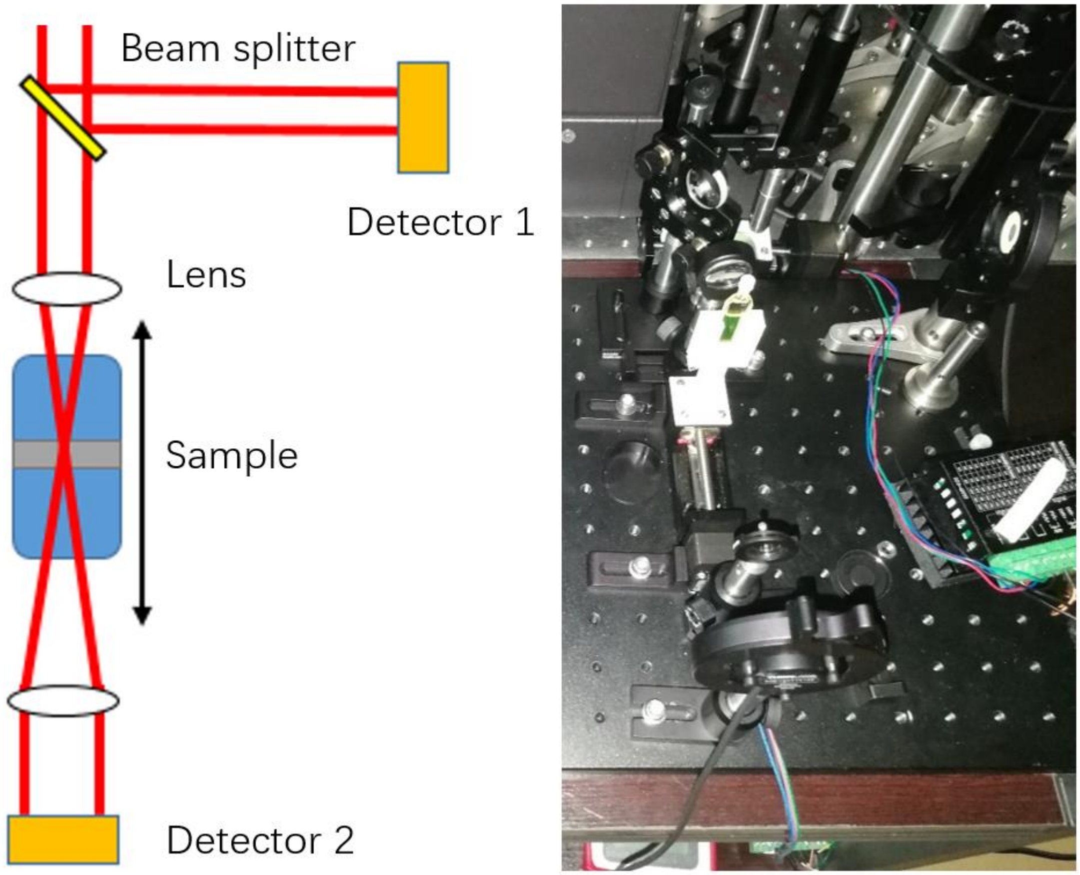

Figure S22. The Schematic and physical drawings of Z-scan experimental techniques.

[1] a) N. S. Makarov, M. Drobizhev, A. Rebane, *Optics Express* **2008**, *16*, 4029-4047; b) A. Karotki, M. Khurana, J. R. Lepock, B. C. Wilson, *Photochem Photobiol* **2006**, *82*, 443-452; c) G. Clay, C. Schaffer, D. Kleinfeld, *The Journal of chemical physics* **2007**, *126*, 025102; d) M. Kauert, P. Stoller, M. Frenz, *Optics express* **2006**, *14*, 8434-8447; e) D. A. Oulianov, I. V. Tomov, A. S. Dvornikov, P. M. Rentzepis, *Proc Natl Acad Sci U S A* **2002**, *99*, 12556-12561; f) R. Sailaja, P. B. Bisht, C. P. Singh, K. S. Bindra, S. M. Oak, *Opt Commun* **2007**, *277*, 433-439.

[2] Y. Guo, X. He, Y. Su, Y. Dai, M. Xie, S. Yang, J. Chen, K. Wang, D. Zhou, C. Wang, *J. Am. Chem. Soc.* **2021**, *143*, 5755-5762.

[3] Z. Wu, B. Ramsundar, Evan N. Feinberg, J. Gomes, C. Geniesse, A. S. Pappu, K. Leswing, V. Pande, *Chemical Science* **2018**, *9*, 513-530.

[4] G. Landrum.

[5] S. M. Lundberg, G. Erion, H. Chen, A. DeGrave, J. M. Prutkin, B. Nair, R. Katz, J. Himmelfarb, N. Bansal, S.-I. Lee, *Nature Machine Intelligence* **2020**, *2*, 56-67.

[6] M. Chołuj, M. M. Alam, M. T. P. Beerepoot, S. P. Sitkiewicz, E. Matito, K. Ruud, R. Zaleśny, *Journal of Chemical Theory and Computation* **2022**, *18*, 1046-1060.
[7] X. Hu, Z. Wang, Y. Su, P. Chen, Y. Jiang, C. Zhang, C. Wang, *Chemistry of Materials* **2021**.
[8] a) F. Terenziani, C. Katan, E. Badaeva, S. Tretiak, M. Blanchard-Desce, *Advanced Materials* **2008**, *20*, 4641-4678; b) T. Lu, F. Chen, *Journal of Theoretical and Computational Chemistry* **2012**, *11*, 163-183.
[9] a) J. S. Murray, P. Politzer, *WIREs Computational Molecular Science* **2017**, *7*; b) J. Zhang, T. Lu, *Physical Chemistry Chemical Physics* **2021**, *23*, 20323-20328.
[10] Z. Liu, T. Lu, Q. Chen, *Carbon* **2020**, *165*, 461-467.
